# Testicular mRNA‐LNP Delivery: A Novel Therapy for Genetic Spermatogenic Disorders

**DOI:** 10.1002/advs.202509855

**Published:** 2026-02-11

**Authors:** Chenwang Zhang, Nan Liang, Wenbo Li, Shuai Xu, Peng Li, Wanze Ni, Na Li, Sha Han, Ningjing Ou, Haowei Bai, Yuxiang Zhang, Furong Bai, Yifan Sun, Dewei Qian, Xinjie Bu, Erlei Zhi, Ruhui Tian, Yuhua Huang, Jingpeng Zhao, Fujun Zhao, Hao Chen, Zheng Li, Chencheng Yao

**Affiliations:** ^1^ Department of Andrology the Center for Men's Health Urologic Medical Center Reproductive Medicine Center Shanghai Key Laboratory of Reproductive Medicine Shanghai General Hospital Shanghai Jiao Tong University School of Medicine Shanghai China; ^2^ State Key Laboratory of Reproductive Medicine and Offspring Health Nanjing Medical University Nanjing China; ^3^ Department of Human Cell Biology and Genetics SUSTech Homeostatic Medicine Institute Joint Laboratory of Guangdong & Hong Kong Universities For Vascular Homeostasis and Diseases School of Medicine Southern University of Science and Technology Shenzhen Guangdong China; ^4^ Taizhou School of Clinical Medicine The Affiliated Taizhou People's Hospital of Nanjing Medical University Nanjing Medical University Nanjing China; ^5^ Department of Urology Department of Interventional Medicine Guangdong Provincial Key Laboratory of Biomedical Imaging the Fifth Affiliated Hospital Sun Yat‐sen University Zhuhai Guangdong China

**Keywords:** genetic defects, male infertility, mRNA‐LNP delivery, restoration of spermatogenesis, spermatogenic disorders

## Abstract

Uniform testicular maturation arrest is a severe form of male infertility characterized by the presence of germ cells that do not complete spermatogenic development. It is usually caused by meiotic arrest with genetic variants and is difficult to treat via drugs or surgery. mRNA‐lipid nanoparticle (LNP) delivery is a promising therapeutic option for maturation arrest with monogenic variants via protein replacement therapy. Herein, a spermatocytes‐tropic LNP (Pool1‐LNP3) was identified via a library of 30 ionizable lipids screening. And in vivo delivery of this novel LNP composition using rete testis microinjection was shown to be high spermatocytes targeting with high transfection efficiency. Thereafter, it was revealed that in vivo delivery of Pool1‐LNP3 encapsulating *Msh5* mRNA could promote crossover formation and restore spermatogenesis in *Msh5^D486Y/D486Y^* mouse models with DNA double‐strand break (DSB) recombination defects. Notably, the offspring without genomic integration was born using intracytoplasmic sperm injection (ICSI) derived from the rescue of *Msh5^D486Y/D486Y^* mouse and embryo transfer. In addition, it was demonstrated that *Maps* mRNA‐LNP3 recovered spermatogenesis in *Maps* KO mouse with meiotic arrest. Altogether, these findings suggested that this spermatocyte‐tropic mRNA‐LNP delivery could become a viable and applicable strategy for the treatment of spermatogenic disorders with genetic defects, providing a foundation for future clinical application.

## Graphical abstract

In the current study, we developed a novel therapy for genetic spermatogenic disorders. It was revealed that in vivo delivery of mRNA LNP3 could restore spermatogenesis in *Msh5^D486Y/D486Y^* and *Maps* KO mouse models with meiotic arrest. Notably, the offspring without genomic integration was born using ICSI derived from the rescue of *Msh5^D486Y/D486Y^* mouse and embryo transfer.

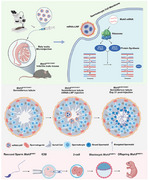

## 1 Introduction

Infertility constitutes a profound global health burden, affecting 10%–15% couples worldwide and contributing to significant psychological distress and socioeconomic impacts [1]. Approximately 50% of infertility‐affected cases involved male factors [2]. And spermatogenic disorders is assumed to be the most severe type. Spermatogenesis is an intricate process orchestrated by over 2000 genes. Spermatogenic disorders could result in non‐obstructive azoospermia (NOA), which is classified as Sertoli cell only syndrome (SCOS), hypo‐spermatogenesis (HS), or mature arrest (MA) according to testis biopsy and subsequent pathologic analysis. Uniform testicular maturation arrest was a subset of NOA, and it was susceptible to genetic defects, including microdeletion of Y chromosome, chromosomal translocation, and monogenic disorder [3, 4]. The application of whole‐exome sequencing (WES) technology has led to the identification of numerous pathogenic variants in essential regulators of spermatogenesis, for instance: MutS Homolog 5 (*MSH5*), Chromosome 3 Open Reading Frame 62 (*C3ORF62*), Meiotic Nuclear Divisions 1 (*MND1*), Meiosis Inhibitor Protein 1 (*MEI1*), and Synaptonemal Complex Protein 2 (*SYCP2*) [5, 6, 7, 8, 9, 10]. These genetic variants are usually associated with defects of the events of meiosis, including synapsis complex formation and DNA double‐strand break (DSB) combination, which leaded into meiotic arrest and NOA [11]. For patients with genetically driven uniform testicular maturation arrest, current endocrine therapies are largely ineffective. In addition, the success rate of sperm retrieval via microdissection testicular sperm extraction (micro‐TESE) remains extremely low [12, 13, 14]. Given the vast heterogeneity of genetic variants implicated in spermatogenic disorders and the current lack of broad‐spectrum therapeutic strategies tailored to distinct genetic etiologies in clinical practice, there is an urgent need to develop a broadly applicable treatment modality capable of addressing diverse genetic variants underlying spermatogenic disorders.

Gene therapies primarily address the genetic defects of disease pathogenesis rather than merely alleviating symptoms. Previous study revealed that CRISPR/Cas9 combined with in vivo electroporation could be used to restore spermatogenesis by correcting the mutant locus in *Msh5^D486Y/D486Y^* mouse model based on pathogenic variants identified in patients with spermatogenic disorder [8]. However, the clinical application of germline gene editing remains constrained by off‐target risks and ethical prohibitions in genome editing of human gametes and zygotes. Consequently, developing alternative therapeutic strategies for diverse pathogenic variants is imperative. Protein replacement therapy (PRT) emerges as a promising approach for treating spermatogenic disorders with genetic defects. mRNA‐based delivery is the widely applicated PRT utilizing carriers to deliver protein‐coding mRNA into target cells for transient translation. Following cellular delivery, mRNA is directly translated into protein in the cytoplasm, where each protein undergoes post‐translational modifications and native folding in vivo to perform its physiological functions accurately. This intrinsic capacity to generate fully functional proteins in situ underscores a critical advantage of mRNA‐based therapeutics over exogenous protein supplementation [15]. Crucially, mRNA therapeutics enable transient protein expression without genomic integration, minimizing mutagenic risks associated with permanent genetic alterations [16, 17]. Recent advancements in chemical modifications (e.g., pseudouridine incorporation) and lipid nanoparticle (LNP) delivery systems have remarkedly improved mRNA stability, translational efficiency, and cell‐type specificity [18]. Thus, mRNA‐based PRT could be used as a particularly suitable strategy for rescuing spermatogenic disorders caused by genetic variants [19].

Microinjection into seminiferous tubules of naked mRNA followed by electroporation has showed partial efficacy in rescue of spermiogenesis defects in *Armc2*‐knockout mouse models [20]. However, the electroporation technique is difficult to master and increases the risk of lesions when poorly performed. It also exhibited suboptimal delivery efficiency, raising substantial concerns regarding safety and reproducibility in human applications. These findings underscore the critical need for advanced delivery platforms to overcome the inherent instability and low efficacy associated with unformulated mRNA therapies. Adeno‐associated virus (AAV) vectors, while efficient in gene delivery, raise concerns over immunogenicity, risk of genomic integration, and limited cargo size [21, 22, 23, 24]. However, lipid nanoparticles validated by their clinical success in COVID‐19 vaccines, have revolutionized nucleic acid delivery by enabling efficient mRNA encapsulation, endosomal escape, and controlled protein expression [25]. Recent advances in mRNA‐LNP have expanded their utility beyond liver‐centric diseases such as lungs, placenta, and brain [26, 27, 28]. However, in the context of treating spermatogenic failure caused by genetic variants, research on mRNA‐LNP delivery remained largely unexplored. Although previous studies have reported that LNP‐mediated delivery of self‐amplifying RNA (saRNA) successfully rescued the meiotic arrest phenotype in *DNA meiotic recombinase 1* (*Dmc1*) knockout (KO) mice, several concerns remain [29]. It has yet to be verified whether the prolonged overexpression of saRNA within germ cells might lead to toxicity, and the in vivo safety of the virus‐derived nonstructural proteins encoded by the saRNA has not been fully established. Moreover, given that the saRNA contains alphavirus replicase genes and encodes an RNA‐dependent RNA polymerase (RdRP) complex with a length approaching 7 kb, delivering larger target gene sequences would undoubtedly pose a significant challenge to this approach.

Herein, we reported the use of an in vivo screening approach to develop an mRNA‐LNP platform to restore spermatogenesis in *Msh5^D486Y/ D486Y^* mouse models derived from NOA‐affected patients [8]. Patients and mice carrying a variant in *MSH5* presented a meiotic arrest phenotype and are infertile. Using a rationally designed LNP library, we identified formulations with enhanced tropism for spermatocytes, achieving transient restoration of functional protein expression without genomic integration. In vivo delivery of Pool1‐LNP3 encapsulating *Msh5* mRNA could promote crossover formation and restore spermatogenesis in *Msh5^D486Y/D486Y^* mouse models with DSB recombination defects. Notably, the offspring without genomic integration was born using intracytoplasmic sperm injection (ICSI) derived from rescue of *Msh5^D486Y/D486Y^* mouse and embryo transfer. Furthermore, no obvious inflammation and histologic damage in any tissue were detected after in vivo delivery of LNP. In addition, it was demonstrated that *Maps* mRNA‐LNP3 recovered spermatogenesis in *Maps* KO mouse with meiotic arrest. Therefore, this approach not only circumvents the limitations of viral vectors but also establishes a framework for repeatable, broad‐spectrum treatment of genetic male infertility. Our findings highlighted the potential of mRNA‐LNPs to overcome therapeutic barriers in clinical reproductive medicine, offering a paradigm shift in addressing spermatogenic disorders with genetic defects.

## 2 Materials and Methods

### 2.1 Mouse Model and Cell Line

All animal care and experiments complied with the guidelines of the National Institutes of Health and were approved by the Animal Care Committee of Shanghai General Hospital (2022AWS0287). The *Maps* KO mouse in a C57BL/6 genetic background was generated by Cyagen Biosciences (Suzhou, China), and a 1.8 kb genomic DNA fragment spanning exon 3 was deleted using the CRISPR/Cas9‐mediated genome‐editing system, the strategy was according to the previous study [30]. The targeted gRNAs and Cas9 protein were co‐injected into the zygotes from C57BL/6 mice to generate targeted knock‐out offspring (gRNA: gRNA‐A1: TCACCAGTACATTTTCGTCTTGG; gRNA‐A2: GTTTAATTCATACCGATGATTGG). The F0 founder mice were identified by PCR and DNA sequencing analysis and were bred with wild‐type mice to obtain F1 heterozygous mutant (*Maps^+/−^*) mice, and the homozygous mice (*Maps^−/−^*) were obtained from breeding pairs of *Maps* ^+/−^ male and female mice. DNA isolated from tail was used for genotyping by PCR and Sanger sequencing as described previously [8, 30], the primers listed in Table S1. All mice were maintained under controlled environmental conditions (temperature: 24 ± 1°C; relative humidity: 50%–60%) with a standard 12‐h light‐and‐dark cycle. The HEK‐293T (RRID: CVCL_0063), TM3 (RRID: CVCL_4326), and TM4 (RRID: CVCL_4327) cells were obtained from American Type Culture Collection, and cultured with Dulbecco's Modified Eagle Medium (DMEM, Gibco, Waltham, MA, USA) supplemented with 10% fetal bovine serum (FBS, HyClone, Marlborough, MA, USA) under standard culture conditions (37°C, 5% CO_2_) without mycoplasma contamination.

### 2.2 Lipid Nanoparticle Synthesis

**FIGURE 1:**
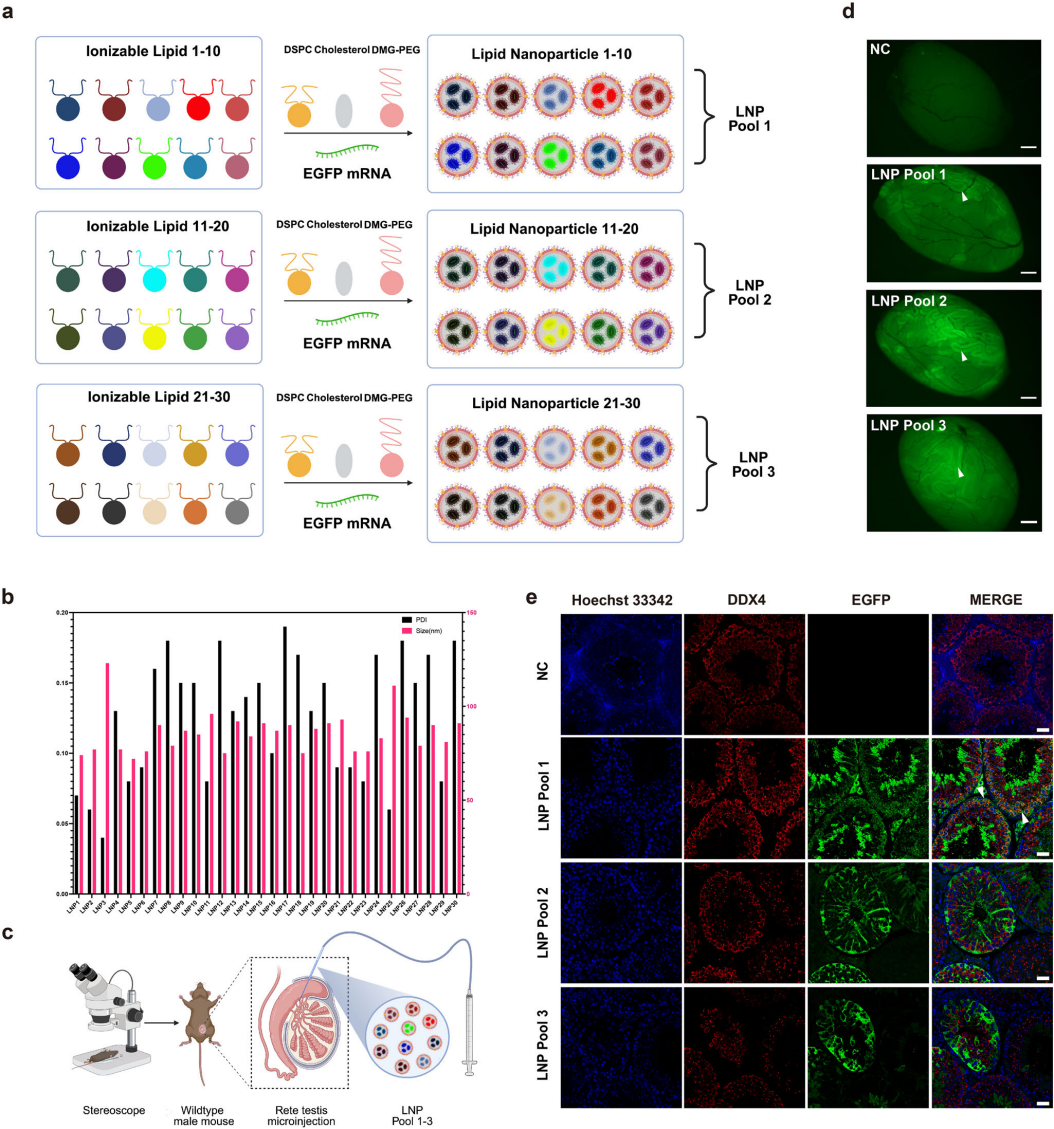
In vivo screening to identify a spermatocyte‐tropic LNP formulation for the restoration of spermatogenic disorders. (a) In vivo screening was employed to identify a spermatocyte‐tropic formulation LNP from a library of total 30 distinct LNP formulations. Thirty LNP formulations were categorized into 3 LNP pools (Pool 1–3), each comprising an equimolar combination of 10 LNPs, (Created by Biorender.com). (b) Size and polydispersity index (PDI) of 30 LNP formulations. (c) Schematic diagram illustrating rete testis microinjection into seminiferous tubules of the testis, (Created by Biorender.com). (d) EGFP expression was observed in the seminiferous tubules 24 h after injection of 3 LNP pools, NC denotes the negative control group administered with PBS (each group n = 3), white arrowheads indicate the EGFP positive seminiferous tubules, scale bar = 5 mm. (e) Immunofluorescence staining of EGFP and the germ cell marker DEAD‐Box Helicase 4 (DDX4) in testicular sections of 3 LNP pools, white arrowheads indicated the EGFP expression in spermatocytes of Pool 1, NC denoted the negative control group administered with PBS (each group n = 3), scale bar = 20 µm.

LNPs were prepared by mixing lipids in an ethanol phase with mRNA in an aqueous phase in two syringe pumps. In brief, the ethanol phase was prepared by solubilizing a mixture of ionizable lipid, cholesterol (AVT Pharmaceutical Tech, Shanghai, China), 1,2‐distearoyl‐sn‐glycero‐3‐phosphocholine (DSPC, AVT Pharmaceutical Tech, Shanghai, China), and 1,2‐dimyristoyl‐rac‐glycero‐3‐methoxypolyethylene glycol‐2000 (DMG‐PEG 2000, AVT Pharmaceutical Tech, Shanghai, China) at a predetermined molar ratio in ethanol (50: 38.5:10:1.5) (Figure 1a). The aqueous phase was prepared in 10 mM citrate buffer (pH 4.0) with enhanced green fluorescent protein mRNA (*EGFP* mRNA, CATUG Biotechnology, Suzhou, China), *Msh5*, and *Maps* mRNA. Syringe pumps were used to mix the aqueous and ethanol phases at a ratio of 3:1. The resulting LNPs were dialyzed against 20 mM Tris in a 100 000 MWCO dialysis tube at 4°C for 4 h, and refreshed in 20 mM Tris buffer, following dialysis at 4°C overnight. A RiboGreen RNA assay (Invitrogen) was used to calculate the nucleic acid encapsulation, and a dynamic light scattering (DLS, Zetasizer, Malvern, UK) was used to measure the size and polydispersity index (PDI) of the LNPs (Figure 1b, Table S2).

### 2.3 In Vivo Luciferase mRNA LNP Delivery

Pool1‐LNP3 encapsulating luciferase mRNA was administered by rete testis microinjection. A 4 µg luciferase mRNA‐LNP 3 was injected into the left testis of each wildtype mouse. On days 1, 3, 5, 7, and 9 after injection, fluorescence imaging was captured using Living Image software on In vivo Imaging System (IVIS, PerkinElmer, Waltham, MA, USA). Ten minutes before imaging, d‐luciferin potassium salt (Thermo Fisher Scientific, Waltham, MA, USA) was injected intraperitoneal (i.p.) to mice at a dose of 150 mg kg^−1^, exposure time: 30 s; Binning: Medium; F/Stop: f/1.

### 2.4 RNA Extraction and mRNA Production

Total RNA was isolated from mouse testicular tissue using TRIzol reagent (Invitrogen, Waltham, MA, USA) following the manufacturer's protocol. RNA integrity and purity were verified using a NanoDrop (Thermo Fisher Scientific, Waltham, MA, USA). Reverse transcription was performed using 1 µg RNA with a First Strand cDNA Synthesis Kit (Thermo Fisher Scientific, Waltham, MA, USA).

For mouse *Msh5* and *Maps* mRNA production, the double‐stranded DNA in vitro transcription (IVT) template was synthesized and cloned into a modified pUC19 plasmid vector containing a T7 RNA polymerase promoter sequence. The plasmids were linearized by BspQI (10 units per 1 µg DNA, 37°C overnight) (R0712S, New England BioLabs, Ipswich, MA, USA) [31] and purified using Monarch PCR & DNA Cleanup spin columns (New England BioLabs, Ipswich, MA, USA) [32]. IVT was performed using a HiScribe T7 mRNA Kit with CleanCap Reagent AG (New England BioLabs, Ipswich, MA, USA) following the manufacturer's protocol, and UTP was all replaced by N1‐methyl‐pseudouridine. RNA was purified using Monarch RNA Cleanup spin columns (New England BioLabs, Ipswich, MA, USA). mRNA product integrity was validated using native agarose gel electrophoresis and the mRNA was stored frozen at −80°C for later use.

### 2.5 DNA Ploidy Assay

DNA ploidy assay was performed as previously described with some modifications [33]. In brief, after the removal of tunica albuginea, testicular tissues were enzymatically digested with 1 mg mL^−1^ collagenase type IV (diluted in DMEM) at 37°C for 5 min. Following centrifugation for 1 min at 100 g, the supernatant was removed. Subsequently, 0.6 mg mL^−1^ trypsin was added to digest the tubules at 37°C for 10 min, and FBS was added for stopping digestion. The suspension was filtered through a 40 µm cell strainer and centrifuged for 10 min at 500 g. Finally, cells were suspended in 1× phosphatic buffer solution (PBS) and labeled with propidium iodide (PI) by 1 µg/1 × 10^5^ cells for 5 min on ice. FACS measurements were performed on BD FACSMelody Cell Sorter. Gate was set as previously reported [33].

### 2.6 In Vivo Rete Testis Microinjection and Preparation of Histologic Sections

Briefly, all mice were anesthetized via i.p. administration of avertin (250 mg kg^−1^) and positioned in dorsal recumbency. Following aseptic preparation, a single incision was made approximately 1.5 cm up to the genitals. The testes were pulled out by holding the fat pad. mRNA‐LNP or PBS was injected into the rete testis using a glass capillary under a stereomicroscope(Figure 1c). The harvested testes were fixed in 4% paraformaldehyde (PFA) for 24 h at 4°C, paraffin‐embedded, and sectioned at 5 µm thickness using a rotary microtome (RM2255, Leica, Wetzlar, Germany). Major organs, including the heart, liver, spleen, lungs, kidney and brain of wildtype mice were collected 24 h post injection and major organs of *Msh5^D486Y/D486Y^* and *Maps* KO mice were collected on day 21 post injection. All the tissues were fixed in PFA for 24 h and paraffin‐embedded, 5 µm sections were generated using a rotary microtome (RM2255, Leica, Wetzlar, Germany).

### 2.7 Immunofluorescence Staining

Before staining, tissue sections were dewaxed in xylene, rehydrated using a gradient series of ethanol solutions, and washed in distilled water. After rehydration, sections were processed for antigen retrieval with 10% sodium citrate (pH 6.0) at 115 °C for 15 min. Non‐specific binding was blocked with 5% normal donkey serum containing 0.3% Triton X‐100 for 2 h at room temperature. Primary antibodies (Table S3) were applied overnight at 4°C, followed by species‐matched Alexa Fluor‐conjugated secondary antibodies (1:1000, Invitrogen, Waltham, MA, USA) for 2 h. Nuclear counterstaining utilized Hoechst 33342 (1:1000, Roche, Basel, Switzerland), with imaging performed on a Leica SP8 confocal system (Leica, Wetzlar, Germany) equipped with LAS X software.

### 2.8 Hematoxylin and Eosin Staining and Hematoxylin‐Eosin‐Saffron (HES) Staining

Tissue sections (including testis, epididymis, heart, liver, spleen, lungs, kidneys, and brain) were dewaxed in xylene and rehydrated through a graded ethanol series followed by distilled water rinses. Sections were immersed in hematoxylin solution for 6 min, briefly differentiated in acid alcohol, and blued in tap water. Subsequently, slides were counterstained in acidified eosin solution for 45 s, dehydrated through ascending ethanol gradients, cleared in xylene, and mounted. Whole‐slide digital images were acquired using a Panoramic SCAN slide scanner (3D Histech). Histopathological evaluation for tissue lesions was performed by the experienced pathologists. For Hematoxylin‐Eosin‐Saffron (HES) Staining: tissue sections were mounted on glass slides, deparaffinized in xylene, and rehydrated using graded alcohols. Nuclei were stained by immersion in hematoxylin solution for one min, followed by rinsing in distilled water. Cytoplasmic differentiation was achieved via a 5 s incubation in 1% acid alcohol. Sections were then blued in running tap water for two min. Cytoplasmic counterstaining was performed by incubating slides in eosin solution for five min, followed by a brief distilled water rinse. For saffron counterstaining, slides were immersed for 15 min in saffron (HY‐D0215, MedChemExpress, Monmouth Junction, NJ, USA) solution (warmed to 35°C–40°C prior to use). Then sections were microscopically examined as recommended following a rapid distilled water rinse. Final dehydration was accomplished by two sequential five min incubations in anhydrous ethanol. Slides were cleared briefly in xylene and cover slipped using neutral gum.

### 2.9 Aniline Blue Staining

Caput spermatozoa chromatin maturity was assessed using the aniline blue staining method (R30404, Yuanye biotechnology, Shanghai, China). Spermatozoa were smeared onto an adhesive‐coated glass slide and air‐dried. The smears were fixed with 4% PFA for 30 min at room temperature, followed by rinsing with distilled water. The smears were then stained with aniline blue staining solution for 5 min at room temperature, rinsed with distilled water, dehydrated through a graded series of ethanol, cleared with xylene, and mounted with neutral gum. Spermatozoa with immature chromatin appeared purple‐blue, while mature sperm appeared light blue or unstained.

### 2.10 Chromomycin A3 Staining

Caput spermatozoa were fixed in 4% PFA, uniformly spread onto glass microscope slides, and overlaid with 100 µL of CMA3 staining solution (0.25 mg mL^−1^ Chromomycin A3 in McIlvaine buffer; (MedChemExpress, Monmouth Junction, NJ, USA) [34]. The McIlvaine buffer (pH 7.0) was prepared with 7 mL of 0.1 M citric acid and 33 mL of 0.2 M Na_2_HPO_4_, supplemented with 10 mM MgCl_2_. Slides were protected from light during a 20 min incubation period, followed by three sequential washes in McIlvaine buffer. Fluorescent signals within individual sperm nuclei were identified using Leica SP8 confocal system (Leica, Wetzlar, Germany) equipped with LAS X software, with 405 nm excitation and brightfield overlay. Spermatozoa were classified into two groups based on fluorescence patterns.

### 2.11 Shorr's Staining

Caput spermatozoa morphology was evaluated using Shorr's staining method (R20357, Yuanye biotechnology, Shanghai, China). Sperm smears were prepared on a glass slide and immediately fixed in 95% ethanol/glacial acetic acid (3:1) fixative solution for 10–15 min. The fixed slides were rehydrated through a graded series of ethanol and rinsed with distilled water. Slides were sequentially stained with hematoxylin, G–6 orange, and EA–50 green, with intermediate rinses and a differentiation step with 1% acetic acid. After staining, the slides were dehydrated, cleared with xylene, and mounted for microscopic examination.

### 2.12 Meiotic Chromosomal Spread Analysis

The tunica albuginea of the mouse testes was removed using microsurgical forceps, then the testes were transferred into 6‐cm dish and washed by PBS. All the tissues were meticulously isolated into single seminiferous tubule using microsurgical forceps and subsequently suspended in hypotonic buffer (30 mM Tris–HCl, 50 mM sucrose, 17 mM trisodium citrate dehydrate, 5 mM EDTA, 2.5 mM dithiothreitol, and 1 mM phenylmethylsulfonyl fluoride) for 25 min. The seminiferous tubules were torn into small pieces and pipetted 15 µL cell suspension spread on a glass slide in a thin layer of PFA solution containing 0.15% Triton X‐100. Slides were placed in a humid chamber and kept for at least 3 h at room temperature. After air drying, the slides were stored at −80°C for further experiments. The slide was then blocked with 5% Bovine Serum Albumin (BSA) and incubated with primary antibodies (Table S3) overnight at 4°C. After washing, the cells were incubated with the secondary antibody (Table S3) for 1 h at room temperature. Images were captured with Leica SP8 confocal system (Leica, Wetzlar, Germany).

### 2.13 Enzyme‐Linked Immunosorbent Assay (ELISA)

Serum samples of wildtype mice were collected 24 h after *EGFP* mRNA‐LNP3 delivery. Mouse IL‐1 beta ELISA Kit (Servicebio, Wuhan, China) was used to evaluate the IL‐1β level in the blood serum following the instructions of the manufacturer.

### 2.14 Western Blot

Testes and other major organs (heart, liver, spleen, lungs, kidney, and brain) were collected from wildtype mice 24 h post‐injection of PBS, 2 µg *EGFP* mRNA‐LNP 3 and 4 µg *EGFP* mRNA‐LNP 3, biological replicates of each group were 3. Tissues were lysed with RIPA Lysis and Extraction Buffer (Thermo Fisher Scientific, Waltham, MA, USA) with 1% protease inhibitor mixture (Thermo Fisher Scientific, Waltham, MA, USA) in accordance with the manufacturer's guidelines. Protein extracts were acquired from the supernatants and were boiled for 10 min, and equal amounts from each sample were loaded onto 10% SDS–PAGE gels for gel electrophoresis (80 V in the stacking gel and 120 V in the resolving gel). After electrophoresis, the proteins in the gels were transferred to polyvinylidene difluoride membranes (Bio‐Rad, Hercules, CA, USA) and blocked using a 5% milk solution for 1 h at room temperature. These membranes were then incubated with primary antibodies (Table S3) at 4°C overnight and subsequently with secondary antibodies according to the protocol provided by the manufacturer. β‐actin was used as the loading control.

### 2.15 ICSI and Embryo Transfer

Female B6D2F1 mice were super‐ovulated by the injection of 5 IU equine chorionic gonadotropin (San‐Sheng Pharmaceutical, Shenyang, China) and followed by 5 IU human chorionic gonadotropin (hCG, San‐Sheng Pharmaceutical, Shenyang, China) 48 h later. Cumulus‐oocyte complexes were collected from oviducts at 14–16 h after hCG injection, and they were placed in HEPES‐buffered CZB medium and treated with 0.1% hyaluronidase (Sigma‐Aldrich, St. Louis, MO, USA) to disperse cumulus cells. Sperm were collected from the testis of *Msh5^D486Y/ D486Y^* male mice (11 weeks old) at 3 weeks after mRNA‐LNP injection and cultured in HEPES‐buffered CZB medium for 15 min. The sperm head was separated from the tail by the application of several Piezo pulses, and the head was then injected into the oocyte according to the method described by Kimura and Yanagimachi [35]. Following micromanipulation, all injected oocytes were cultured in G1‐plus (Vitrolife, Gothenburg, Sweden) under standard culture conditions. Pronucleus formation was checked at 6 h after ICSI, and outcomes were scored up to the blastocyst stage. For embryo transfer, two‐cell embryos were surgically transferred into the oviduct of each 8‐week‐old pseudo‐pregnant female. In this study, pseudo‐pregnant females were generated by mating them with sterile males that had undergone bilateral vasectomy [36]. The surgical procedure for generating vasectomized males was carried out as follows: Mature male mice were anesthetized and placed on a warming pad. After aseptic preparation of the surgical site, a small midline incision (∼0.5–1.0 cm) was made in the lower abdomen, just superior to the scrotum. Through this incision, the vas deferens on each side was sequentially identified and carefully separated from the accompanying blood vessels by blunt dissection. Each vas deferens was then permanently occluded by double ligation with silk sutures, followed by transection between the ligatures. After confirming hemostasis, the testes were returned to the abdominal cavity, and the skin incision was closed with sutures. Postoperatively, the males were allowed to recover for at least two weeks to ensure complete clearance of residual sperm before being used to induce pseudo‐pregnancy. Sterility was confirmed by their ability to form vaginal plugs in females without resulting in pregnancy.

### 2.16 Genotyping of Single Sperm and Single Blastocyst

Genotyping of single sperm and single blastocyst was performed using a Multiple Annealing and Looping‐Based Amplification Cycles (MALBAC) WGA kit (XK‐028‐24, Yikon, Suzhou, China) following the manufacturer's protocol. Briefly, individual sperm or blastocysts were lysed in a 5 µL reaction mixture containing 4.5 µL lysis buffer and 0.5 µL lysis enzyme. The samples were incubated at 50°C for 20 min, followed by enzyme inactivation at 80°C for 10 min. To avoid sample loss, cell suspensions were not vortexed after transfer and were briefly centrifuged. For amplification, 62 µL of reaction mix (60 µL processing buffer and 2 µL processing enzyme per sample) was added to the lysate. Thermal cycling was performed under the following conditions: initial denaturation at 94°C for 3 min; 8 cycles of 20 s at 10°C, 30 s at 30°C, 40 s at 50°C, 2 min at 70°C, and 20 s at 95°C; followed by 17–21 cycles (17 cycles for single blastocysts, 19–21 cycles for single sperm cells) of 20 sec at 94°C, 15 s at 58°C, and 2 min at 72°C; and a final extension at 72°C for 5 min. Cycle numbers were optimized based on starting material quantity, as recommended for low‐input samples. Amplified products were stored at 4°C until downstream genotyping analysis.

For genotyping, thermal cycling was performed under the following conditions: initial denaturation at 94°C for 3 min; 35 cycles of 30 s at 94°C, 30 s at 60°C, 30 s at 72°C, and a final extension at 72°C for 5 min. Genotyping strategy was described in the method ‘mice models’ as described above.

### 2.17 Statistical Analyses

**FIGURE 2:**
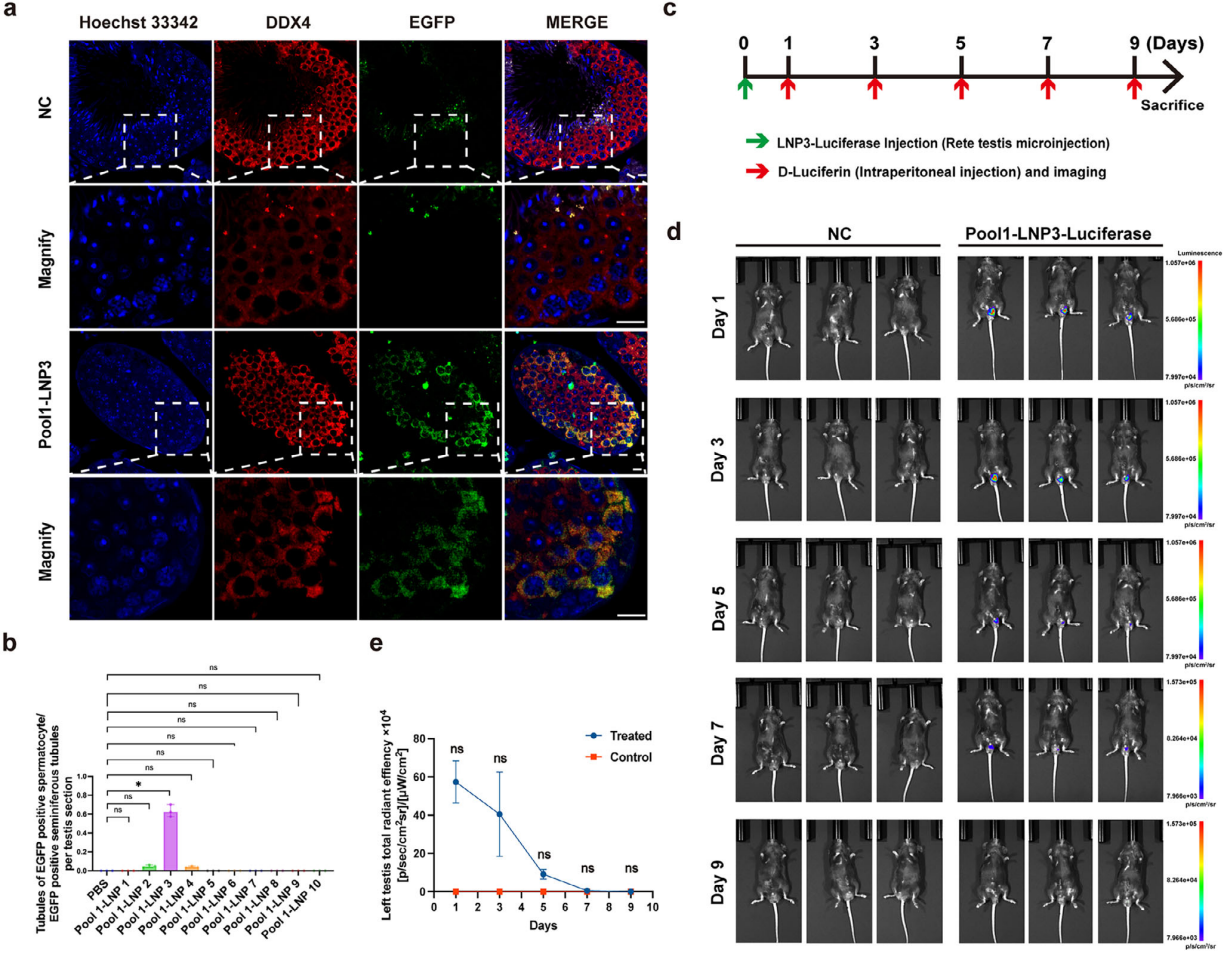
Timeline of biodistribution and expression of mRNA‐LNP3. (a) Immunofluorescence staining of EGFP expression in testis sections after delivery of *EGFP* mRNA‐LNP3, NC denoted the negative control group administered with PBS (each group n = 3), scale bar = 20 µm. (b) The percentage of seminiferous tubules containing EGFP‐positive spermatocytes relative to the EGFP‐positive seminiferous tubule population, intergroup comparisons among the three groups were performed using the non‐parametric Kruskal–Wallis test. Where the overall test indicated statistical significance, pairwise post hoc comparisons were conducted with Bonferroni‐adjusted *p* values to control for type I error. Data were represented as median and interquartile range (IQR), n = 3 biologically independent mice per group. Significance thresholds: ^*^ *p* < 0.05, ns: no significance. (c) Schematic diagram of 4 µg firefly luciferase mRNA‐LNP3 delivery via rete testis microinjection, imaging were performed followed by intraperitoneal injection of d‐luciferin potassium on day 1, 3, 5, 7, 9. (d,e) In vivo mRNA delivery efficacy of Pool 1‐LNP 3 were measured by the IVIS imaging system and expression for 7 days, Mann‐Whitney U test data are represented as median and interquartile range (IQR), significance thresholds: ns: no significance. n = 3 biologically independent mice per group.

**FIGURE 3:**
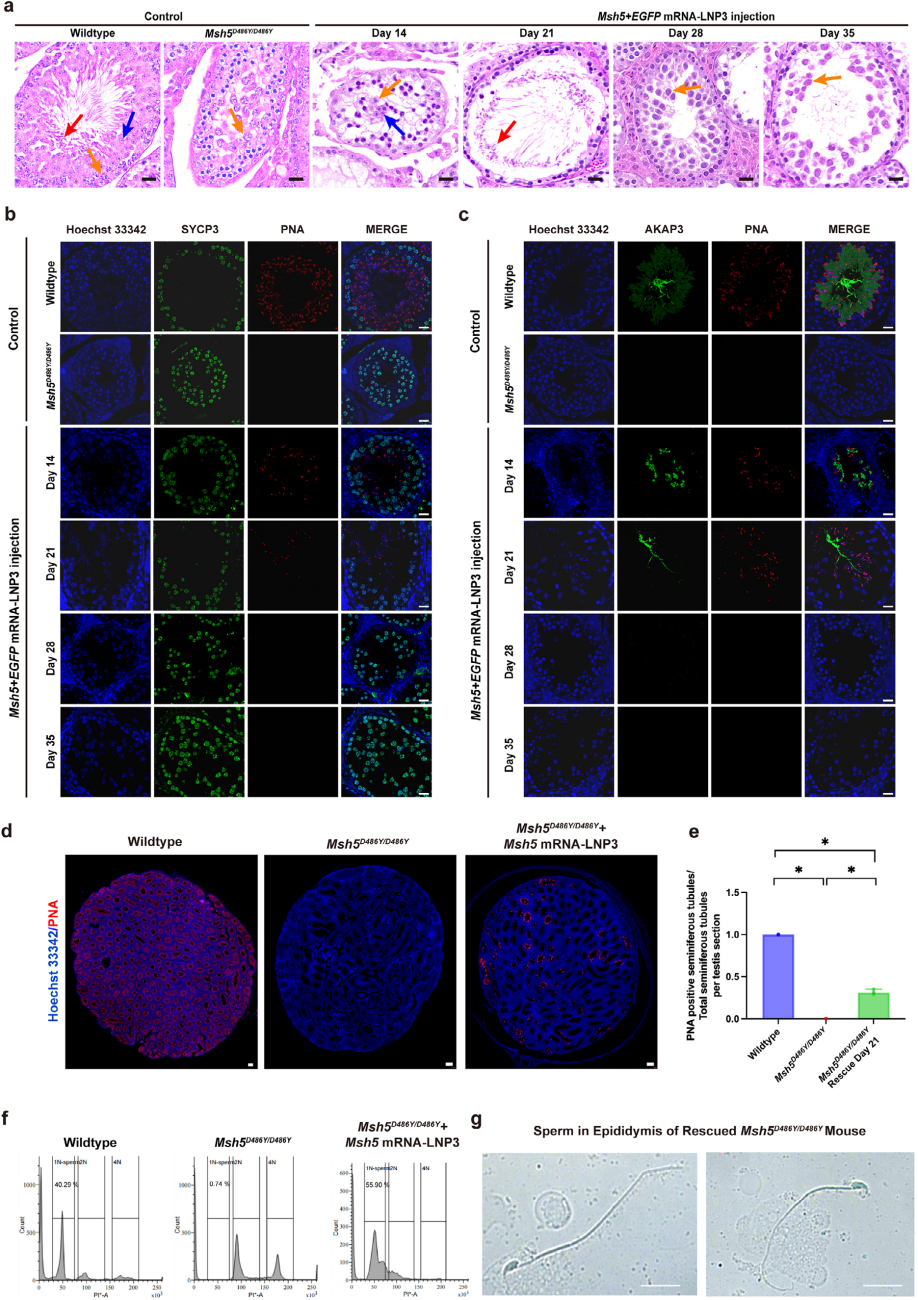
The restoration of spermatogenesis in *Msh5^D486Y/D486Y^* male mice. (a) H&E staining of *Msh5^D486Y/D486Y^* testis sections harvested on day 14, 21, 28, and 35 after injection of *Msh5* mRNA+*EGFP* mRNA‐LNP3 revealed the emergence of elongated spermatids in day 21 and diminished in day 28, yellow arrow indicated spermatocytes, blue arrow indicated round spermatids, red arrow indicated elongated spermatids, scale bar = 20 µm. (b,c) Immunofluorescence staining showed the expression of SYCP3, PNA, and AKAP3 in *Msh5^D486Y/D486Y^* testis sections harvested on day 14, 21, 28, and 35 after injection of *Msh5* mRNA+*EGFP* mRNA‐LNP3, scale bar = 20 µm. (d) Immunofluorescence staining showed the expression of PNA in whole testicular section from wildtype, *Msh5^D486Y/D486Y^* and *Msh5^D486Y/D486Y^* mice on day 21 after injection of *Msh5* mRNA+*EGFP* mRNA‐LNP3, scale bar = 100 µm. (e) Comparison of the proportion of PNA positive seminiferous tubules in wildtype, *Msh5^D486Y/D486Y^* and *Msh5^D486Y/D486Y^* rescued testis, intergroup comparisons among the three groups were performed using the non‐parametric Kruskal–Wallis test. Where the overall test indicated statistical significance, pairwise post hoc comparisons were conducted with Bonferroni‐adjusted *p* values to control for type I error. Data were represented as median and interquartile range (IQR), n = 3 biologically independent mice per group. Significance thresholds: ^*^ *p* < 0.05. (f) Comparison of the proportion of haploid germ cells by flow cytometry staining with propidium iodide (PI). (g) Morphology of sperm from epididymis in day 28 after injection, scale bar = 10 µm.

**FIGURE 4:**
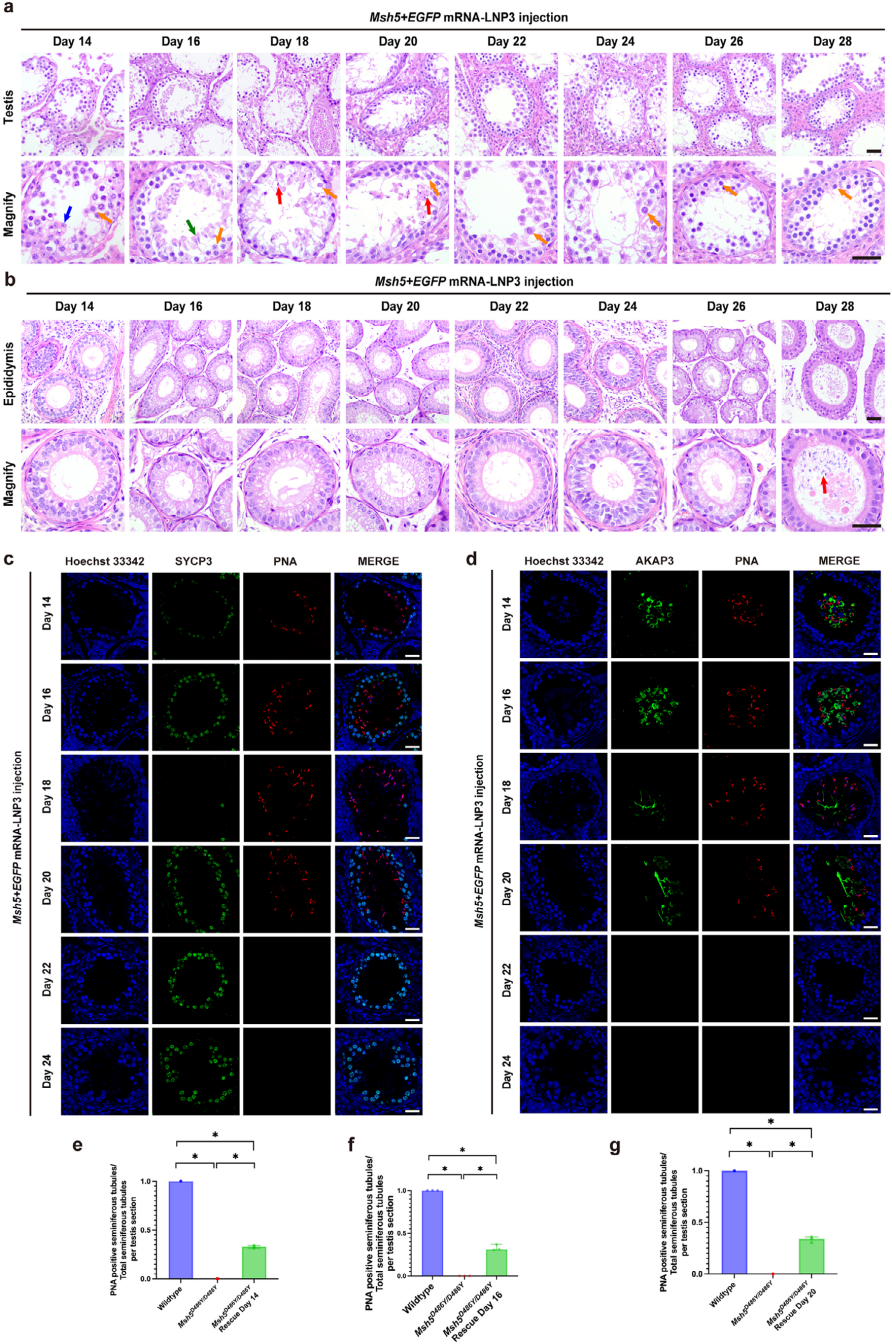
Timeline of restoration of spermatogenesis in *Msh5^D486Y/D486Y^* mice. (a,b) Hematoxylin and eosin staining of *Msh5^D486Y/D486Y^* testis and caput epididymis sections harvested in 14, 16, 18, 20, 22, 24, 26, and 28 days after injection of *Msh5* mRNA+*EGFP* mRNA‐LNP3, yellow arrow indicated spermatocytes, blue arrow indicated round spermatids, green arrow indicated elongating spermatids and red arrow indicated elongated spermatids, scale bar = 50 µm. (c,d) Immunofluorescence staining showed the expression of SYCP3, AKAP3, and PNA in *Msh5^D486Y/D486Y^* testis sections harvested in 14, 16, 18, 20, 22, and 24 days after injection of *Msh5* mRNA+*EGFP* mRNA‐LNP3, scale bar = 20 µm. (e–g) Comparison of the proportion of PNA positive seminiferous tubules in wildtype, *Msh5^D486Y/D486Y^* and *Msh5^D486Y/D486Y^* rescued testis post injection day 14, 16 and 20, intergroup comparisons among the three groups were performed using the non‐parametric Kruskal–Wallis test. Where the overall test indicated statistical significance, pairwise post hoc comparisons were conducted with Bonferroni‐adjusted *p* values to control for type I error. Data were represented as median and interquartile range (IQR), n = 3 biologically independent mice per group. Significance thresholds: *p < 0.05.

Statistical analyses were performed using SPSS Statistics 24. Mann‐Whitney U test was used for the results in Figure 2e. For the results in Figures 2, 3, 4, 6, 7d, Figures S1d–f and S2e, intergroup comparisons among the three groups were conducted using the non‐parametric Kruskal–Wallis test. When the overall test indicated statistical significance, pairwise post hoc comparisons were conducted with Bonferroni‐adjusted *p* values to control for type I error. Data were represented as median and interquartile range (IQR), n ≥ 3 biologically independent replicates per group. Statistical significance was defined as ^*^ *p* < 0.05, ^**^ *p* < 0.01, and ns indicating no significant difference.

## 3 Results

### 3.1 Identification of In Vivo Spermatocyte Tropic LNP

Currently, testicular mRNA LNP delivery lacks efficient, safe, and germ cell‐specific vehicles. To address these challenges, we constructed a combinatorial LNP library comprising 30 distinct formulations with different ionizable lipid architectures. Each LNP formulation was systematically prepared with four essential components: ionizable lipid, cholesterol, DSPC, and DMG‐PEG‐2000 with 50: 38.5: 10: 1.5 molar ratio, encapsulating enhanced green fluorescent protein (EGFP)‐encoding mRNA (Figure 1a). A comprehensive analysis was performed to characterize physicochemical properties of the LNP, including size (nm) and PDI (Figure 1b).

All 30 LNPs were divided into three combinatorial pools (Pool 1–3) and transfected into three kinds of cell lines (human embryonic kidney cell line (HEK293T), mouse Sertoli cell line (TM4), and mouse Leydig cell line (TM3) to confirm their expression of EGFP (Figure S1a–c). EGFP was expressed in 3 cell lines after delivery of three LNP pools, but with different efficiency (Figure S1d–f). To evaluate the delivery efficiency in vivo, LNP of three pools were separately injected to the seminiferous tubules of adult wildtype male mice via rete testis microinjection (Figure 1c). Mice were euthanized after 24 h of injection, and testicular wholemount fluorescence imaging confirmed EGFP expression across seminiferous tubules in all three LNP pools (Figure 1d). To delineate the cellular specificity of LNP‐mediated mRNA delivery, testicular tissues underwent immunofluorescence staining with germ cell specific markers DEAD‐Box Helicase 4 (DDX4), which was highly expressed in the cytoplasm of spermatocytes [37, 38]. The results showed that LNP of Pool1 emerged as the only candidate presenting preferential mRNA delivery to germ cells (Figure 1e). To further identify the spermatocyte tropic LNP, we injected all 10 LNPs of Pool1 individually to the seminiferous tubules of wildtype male mice. Immunofluorescence staining confirmed that Pool1‐LNP3 (hereafter referred to as LNP3) exclusively targeted spermatocytes (Figure 2a,b, Figure S2e), and it was shown that EGFP was highly expressed after delivery of EGFP mRNA‐Pool1‐LNP3 in 293T cells (Figure S2a). The chemical structure of the ionizable lipid used for the preparation of LNP3 is shown in Figure S3. However, EGFP was mainly expressed in Sertoli cells after injection of other LNPs of Pool1 (Figure S2b–d). To further assess the duration of intracellular mRNA expression following mRNA delivery, we encapsulated luciferase‐encoding mRNA within LNP3 and injected it into the seminiferous tubules via rete testis microinjection. Images were recorded on days 1, 3, 5, 7, and 9 after injection by an in vivo imaging system (Figure 2c). It was showed sustained luciferase activity from day 1 to day 7 after injection (Figure 2d,e). Altogether, these results demonstrated that LNP3 were superior vehicles for mRNA delivery into spermatocytes and the sustained expression could be effective for the following rescue studies.

### 3.2 Delivery of *Msh5* mRNA Using LNP3 Restored Spermatogenesis in *Msh5^D486Y/D486Y^* Mice With Spermatogenic Disorders

To evaluate whether spermatogenic disorders could be rescued via the novel mRNA delivery strategy, *Msh5^D486Y/D486Y^* male mice with meiotic arrest as described as previously was chosen in the current study [8]. *Msh5* mRNA and *EGFP* mRNA were encapsulated in LNP3 with 1:1 mass ratio and injected to *Msh5^D486Y/D486Y^* male mice (Figure S4a). Testicular wholemount fluorescence imaging on days 7, 14, and 21 after injection revealed the expression of EGFP sustained in the seminiferous tubules for more than 14 days (Figure S4b). Hematoxylin and eosin staining revealed that while spermatogenesis was arrested at the spermatocyte stage in untreated *Msh5^D486Y/ D486Y^* testis, elongated spermatids occurred in the seminiferous tubules by day 21 and disappeared on day 28 after delivery (Figure 3a). Furthermore, we performed immunofluorescence staining for germ cell markers of each stage, including SYCP3 (spermatocyte marker), AKAP3 (spermatid marker), TNP1 (elongating spermatid marker), and peanut agglutinin (PNA)‐lectin to label the acrosome of the spermatid. The results confirmed the presence of PNA‐positive round spermatids as early as day 7 post‐treatment, albeit in low numbers (Figure S4c). By day 14 AKAP3‐positive spermatids had increased significantly in the seminiferous tubules, and by day 21, most spermatids in the tubules had completed spermiogenesis (Figure 3b,c, Figure S4d). However, elongated spermatids were absent in the testes collected on days 28 and 35 after injection, indicating transient expression of the mRNA within spermatocytes, sufficient to facilitate meiotic progression (Figure 3b,c).

To evaluate the therapeutic rescue efficiency of mRNA‐LNP3 delivery, we quantified seminiferous tubules with PNA‐positive spermatids at 21 days after injection. The results revealed that 31.91 ± 2.97% of tubules cross‐sections exhibited PNA‐positive spermatids within their luminal compartments, indicating restored spermatogenesis in these seminiferous tubules (Figure 3d,e). Flow cytometric quantification of testicular cell suspensions showed 55.9% of the testicular cells were haploid germ cells after mRNA delivery (1N population), demonstrating significant rescue efficiency compared with untreated *Msh5^D486Y/ D486Y^* testis (Figure 3f). Furthermore, cytologic examinations revealed that few spermatozoa in the caput epididymis at 28 days after injection showed non‐progressive motility (Movie S1). Also, CMA3 staining, aniline blue staining, Shorr's staining, and PNA staining revealed normal head condensation, intact acrosomal structures, and typical flagellar architecture (Figure 3g, Figure S5).

In order to delineate the critical window for presence of elongated spermatids, testis and caput epididymidis were collected on days 14, 16, 18, 20, 22, 24, 26, and 28 after mRNA delivery. H&E, immunofluorescence and HES staining analysis indicated the presence of elongated spermatids in the seminiferous tubules by day 18 and were no longer detectable by day 22 (Figure 4a,c,d, Figures S6 and S7), whereas H&E staining analysis revealed that there were no spermatozoa until day 28 after injection in the epididymis (Figure 4b). The percentages of PNA‐positive seminiferous tubules at 14, 16, and 20 days after injection were 32.89 ± 1.44%, 32.91 ± 3.57%, and 33.47 ± 2.94%, respectively (Figure 4e–g).

**FIGURE 5:**
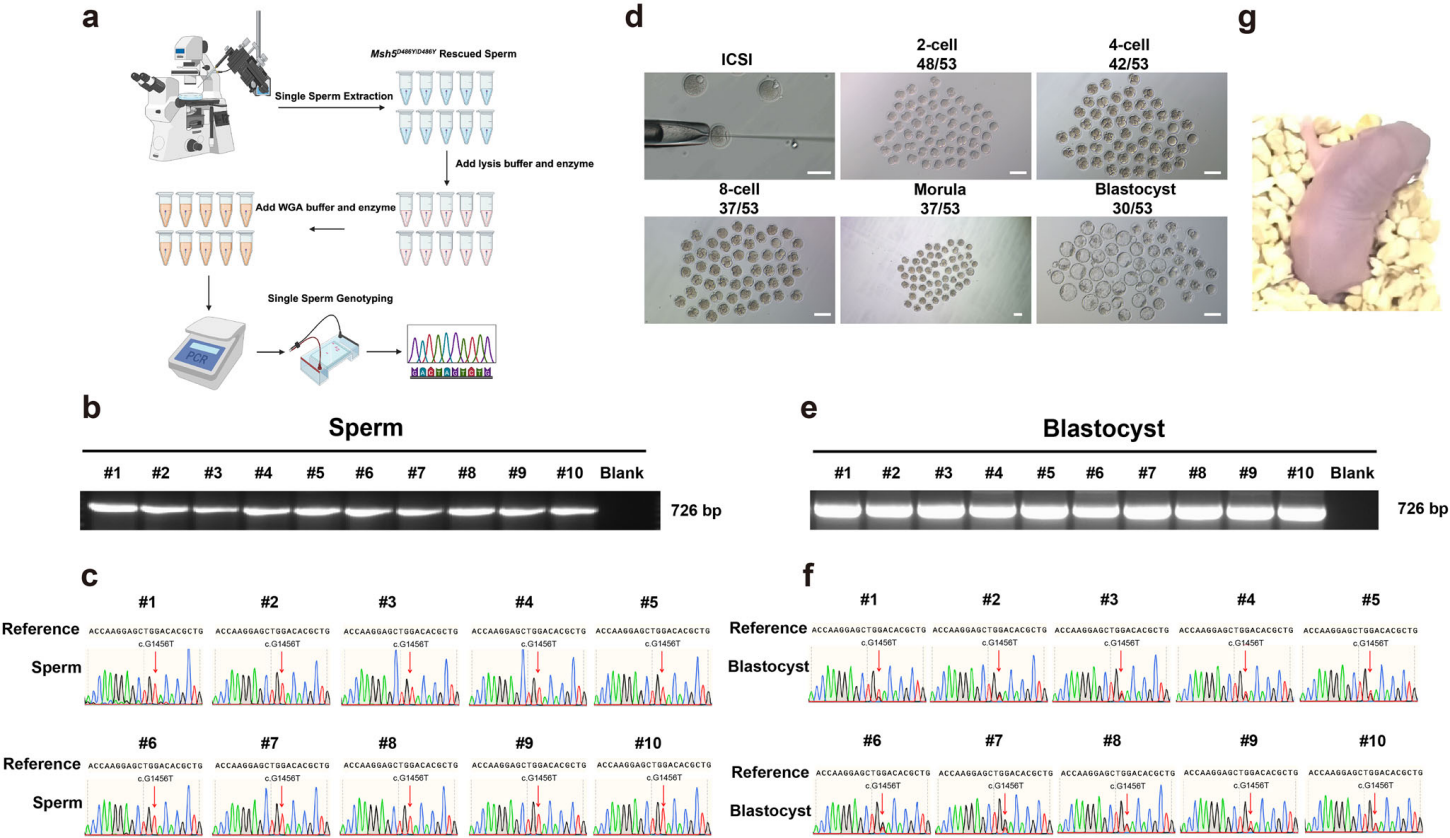
*Msh5* mRNA‐LNP3 restored fertility in *Msh5^D486Y/D486Y^* mice and produced offspring. (a) Schematic diagram of single sperm genotyping of *Msh5* mRNA‐LNP3 rescued sperm, (Created by Biorender.com). (b,c) Genotyping of the randomly chosen 10 single sperm from the rescued testis, Sanger sequencing confirmed the variant of *Msh5* (c.G1456T). (d) Representative images of two‐cell embryos, four‐cell embryos, eight‐cell embryos, morula embryos, and blastocyst embryos after ICSI of the *Msh5* mRNA‐LNP3 rescued sperm, scale bar = 100 µm. (e,f) Genotyping of the randomly chosen 10 blastocysts, Sanger sequencing confirmed the heterozygous variant of *Msh5* (c.G1456T) in the blastocysts. (g) Offspring (F1) obtained using sperm from rescued *Msh5^D486Y/D486Y^* male mice by ICSI.

To evaluate whether the delivered mRNA integrated into rescued sperm genome, we collected the sperms from the testis on day 20 after injection and performed genotyping on 10 randomly selected sperm (Figure 5a), Sanger sequencing revealed that all 10 sperm samples exhibited a mutation genotype (*Msh5* p.D486Y) (Figure 5b,c). To further evaluate the fertility of the mice after *Msh5* mRNA‐LNP3 delivery, we microinjected 53 sperms into wildtype female mouse oocytes and observed 48 embryos developed to the 2‐cell stage on day 2 after ICSI. On day 5–6 after ICSI, we observed 37 embryos proceeded to morula stage and 30 embryos proceeded to blastocysts (Figure 5d). Sanger sequencing revealed that all 10 randomly chosen blastocysts exhibited heterozygous genotypes (*Msh5* p.D486Y) (Figure 5e,f). After transplantation of the embryos, a live offspring was successfully delivered (Figure 5g). This genetic pattern provides conclusive evidence that the therapeutic mRNA delivery restored the full development of spermatogenesis in *Msh5^D486Y/D486Y^* male mice without genomic editing, highlighting the therapeutic potential of mRNA‐based approaches in the treatment of spermatogenic disorders with genetic defects.

### 3.3 Delivery of *Msh5* mRNA Using LNP3 Rescued the Meiotic Defects in *Msh^5D486Y/D486Y^* Mice

To decipher the mechanism of restoration of spermatogenesis in *Msh5^D486Y/D486Y^* mice after *Msh5* mRNA LNP3 delivery, meiotic chromosomal spread analysis was performed to evaluate the meiotic progression and recombination. In wildtype DMC1, foci were abundant during early prophase I and progressively diminished in pachytene, indicating timely strand invasion and homologous recombination progression. In contrast, DMC1 foci of *Msh5^D486Y/D486Y^* spermatocytes persisted into pachytene, suggesting defects in strand invasion, intermediate resolution, and a prolonged recombination process. However, after injection of mRNA‐LNP3, the rescued spermatocytes showed normal DSB formation and repair process by timely diminished DMC1 foci in pachytene (Figure S8a). Furthermore, the result of chromosomal spread analysis by SYCP1 and SYCP3 indicated both the *Msh5^D486Y/D486Y^* and the rescued spermatocytes could form a normal synaptonemal complex (Figure S8b).

The mismatch repair protein MLH1 was used to mark the formation of crossovers during meiotic recombination [39]. In mid‐pachytene spermatocytes, MLH1 foci appeared as ∼22 discrete foci per nucleus, with at least one per homolog pair, evenly distributed along the synapsed axes [40]. The result of chromosomal spread analysis showed almost no MLH1 foci in *Msh5^D486Y/D486Y^* pachytene spermatocytes, which is consistent with previous studies [8], indicating the elimination of crossovers. In contrast, the rescued group displayed a significantly increased number of MLH1 foci, suggesting successful restoration of crossover formation (Figure S8c). Altogether, these results suggested that delivery of *Msh5* mRNA LNP3 could rescue the DSB combination defects and meiotic arrest in *Msh5^D486Y/D486Y^* pachytene spermatocytes.

### 3.4 Safety of *Msh5* mRNA‐LNP3 Delivery via Rete Testis Microinjection In Vivo

**FIGURE 6:**
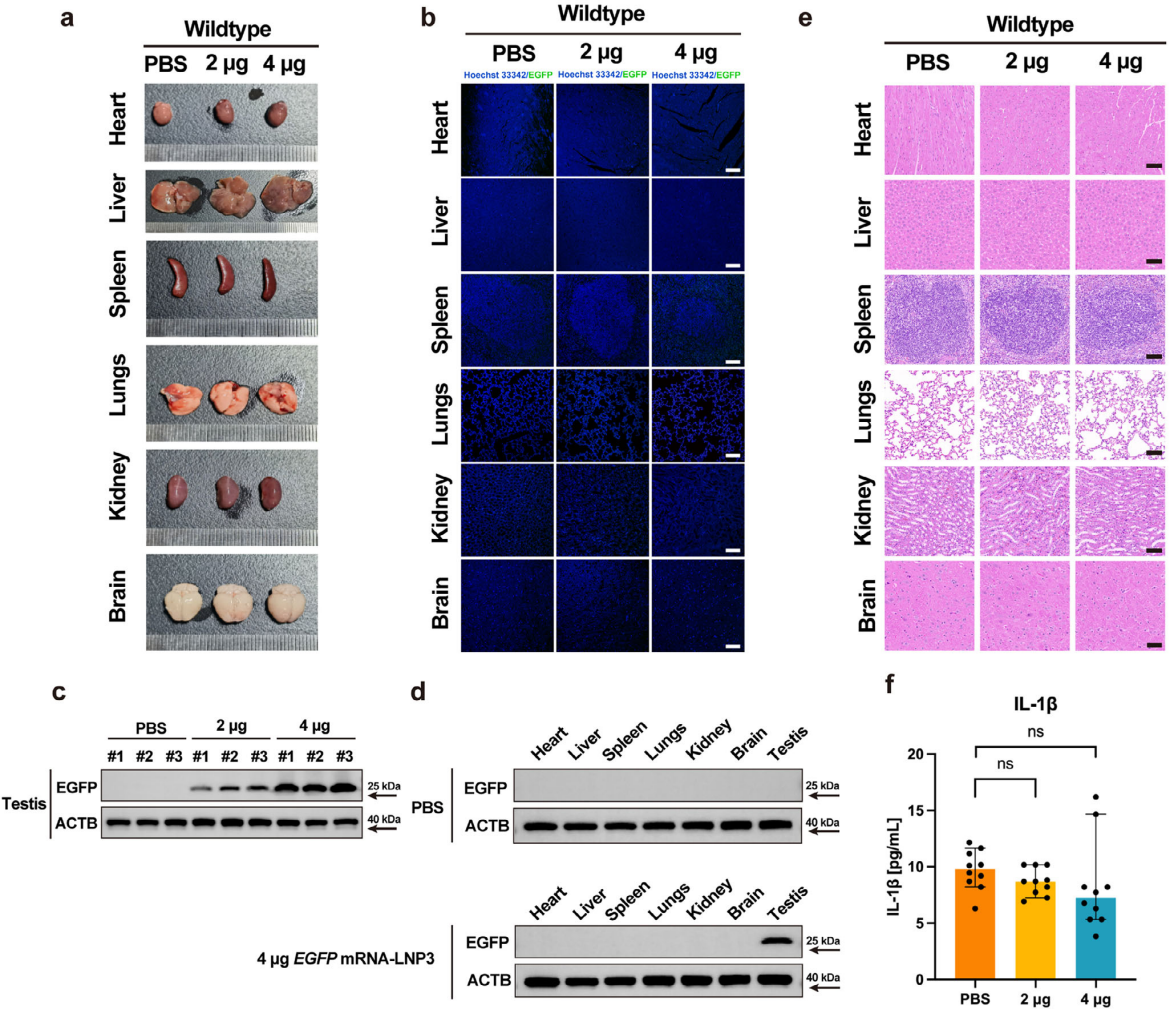
Systemic safety validation of LNP3 and payload. (a) No macroscopic lesions in major organs (heart, liver, spleen, lungs, kidney and brain tissue) following injection of varying *EGFP* mRNA‐LNP3 doses (2 and 4 µg per testis) in wildtype mice, samples were collected 24 h after injection, n = 3 biologically independent mice per group. (b) Immunofluorescence staining revealed no ectopic expression of EGFP in major organs (heart, liver, spleen, lungs, kidney, and brain tissue) following injection of varying *EGFP* mRNA‐LNP3 doses (2 and 4 µg per testis) in wildtype mice, scale bar = 50 µm, n = 3 biologically independent mice per group. (c) Western blot analysis showed the expression of EGFP in testicular samples from wildtype testis following injection of varying *EGFP* mRNA‐LNP3 doses (2 and 4 µg per testis) and PBS as negative control, n = 3 biologically independent mice per group. (d) Western blot analysis showed the expression of EGFP in major organ samples (heart, liver, spleen, lungs, kidney and brain tissue) from wildtype mice following injection of *EGFP* mRNA‐LNP3 high doses 4 µg per testis and PBS, n = 3 biologically independent mice per group. (e) Hematoxylin and eosin staining analysis showed preserved tissue architecture without significantly pathological alterations in major organs (heart, liver, spleen, lungs, kidney, and brain), scale bar = 50 µm, n = 3 biologically independent mice per group. (f) ELISA assay showed the expression of IL‐1β in the blood after injection of varying *EGFP* mRNA‐LNP3 doses (2 µg per testis and 4 µg per testis), intergroup comparisons among the three groups were performed using the non‐parametric Kruskal–Wallis test. Where the overall test indicated statistical significance, pairwise post hoc comparisons were conducted with Bonferroni‐adjusted *p* values to control for type I error. Data were represented as median and interquartile range (IQR), n = 10 biologically independent mice per group. Significance thresholds: ns: no significance.

To confirm the safety of *Msh5* mRNA‐LNP delivery in vivo, we tested the safety of Pool1‐LNP3 and *Msh5* mRNA in vivo, respectively. First, we delivered PBS, 2 and 4 µg *EGFP* mRNA‐LNP3 into seminiferous tubules of wildtype mice via rete testis microinjection to explore the safety of LNP. And multiple tissues throughout the body were collected, including the testis, blood, heart, liver, spleen, lungs, kidneys, and brain after 24 h. Gross photograph showed no significant abnormalities in any organs assessed (Figure 6a). Also, immunofluorescence analysis revealed that *EGFP* mRNA did not leak into other major organs throughout the body after rete testis injection (Figure 6b). Western blot revealed that EGFP was expressed in testis after mRNA‐LNP delivery via concentration‐dependent manner, and no expressions of EGFP except testis were detected in multiple tissues throughout the body (Figure 6c,d). Furthermore, H&E staining revealed no significant abnormalities in any organs assessed (Figure 6e). ELISA assay showed there were no significant differences of the expression of pro‐inflammatory cytokines (IL‐1β) among the three groups (PBS, 2 and 4 µg, respectively) (Figure 6f).

Subsequently, we evaluated the safety of *Msh5* mRNA in vivo via rete testis microinjection of a mixture of *Msh5* mRNA and *EGFP* mRNA at 2 µg in *Msh5^D486Y/ D486Y^* male mice. Multiple tissues throughout the body on day 21 after injection were collected, including heart, liver, spleen, lungs, kidney and brain. And the histological analysis showed that there were no significant abnormalities in any organs assessed compared with untreated *Msh5^D486Y/D486Y^* male mice (Figure S9). Collectively, these results illustrated that *Msh5* mRNA‐LNP3 delivery via rete testis microinjection in vivo showed no obvious toxicity in multiple tissues throughout the body.

### 3.5 mRNA‐LNP Delivery Restored Spermatogenic Disorders in *Maps* KO Mice: Toward Versatile Therapy

**FIGURE 7:**
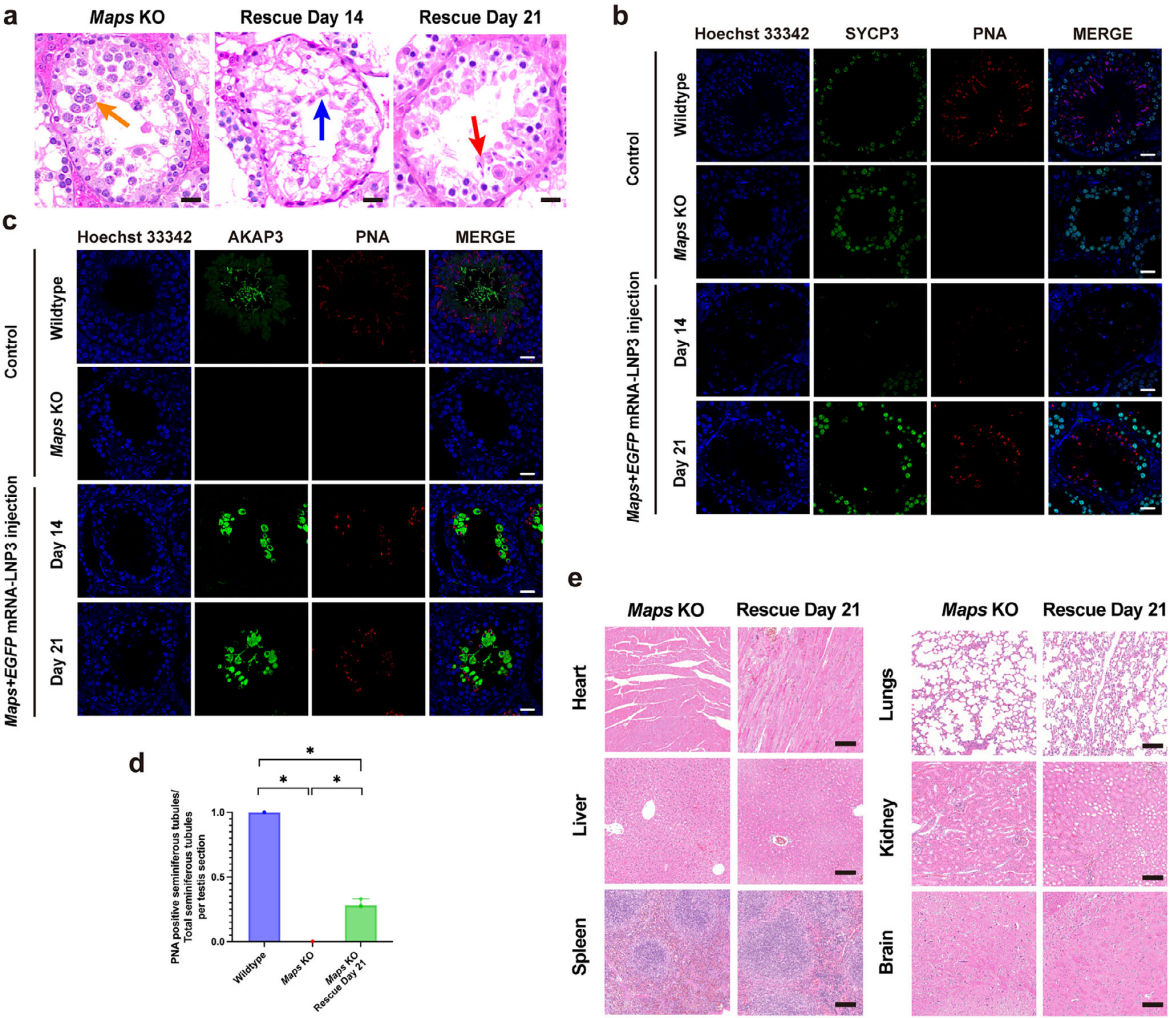
Restoration of spermatogenesis in *Maps* KO male mice. (a) Hematoxylin and eosin staining revealed the appearance of round spermatids and elongated spermatids on day 14 and 21 after injection respectively, yellow arrow indicated spermatocytes, the blue arrow indicated round spermatids, red arrow indicated elongated sperm, scale bar = 20 µm. (b,c) Immunofluorescence staining showed the expression of SYCP3, PNA, and AKAP3 in *Maps* KO testis sections harvested on day 14 and 21 after injection of *Maps* mRNA+*EGFP* mRNA‐LNP3, scale bar = 20 µm. (d) Comparison of the proportion of PNA positive seminiferous tubules in wildtype, *Maps* KO, and *Maps* KO rescued testis at day 21 after injection, intergroup comparisons among the three groups were performed using the non‐parametric Kruskal–Wallis test. Where the overall test indicated statistical significance, pairwise post hoc comparisons were conducted with Bonferroni‐adjusted *p* values to control for type I error. Data were represented as median and interquartile range (IQR), n = 3 biologically independent mice per group. Significance thresholds: ^*^ *p* < 0.05. (e) Hematoxylin and eosin staining analysis showed preserved tissue architecture without significantly pathological alterations in major organs (heart, liver, spleen, lungs, kidney, and brain) in *Maps* KO mice at 21 days after injection, scale bar = 50 µm, n = 3 biologically independent mice per group.

To explore the broad applicability of mRNA‐LNP technology, *Maps* (*C3orf62*) KO mice via CRISPR/Cas9 mediated genome editing were generated as described previously [30]. *Maps* KO mice faithfully recapitulated the human infertility phenotype as we reported previously, showing complete spermatogenic arrest at the spermatocyte stage. Intriguingly, sequential restoration of spermatogenesis was observed after *Maps* mRNA+*EGFP* mRNA LNP3 (2 µg per testis) delivery via rete testis microinjection. Specifically, seminiferous tubules exhibited the occurrence of round spermatids on day 14 after injection and elongated spermatids on day 21 after injection (Figure 7a). The results of immunofluorescence staining also revealed that the PNA‐positive round spermatids and AKAP3‐positive elongating spermatids could be detected on day 14 post injection and TP1‐positive elongated spermatids emerged on day 21 (Figure 7b,c, Figure S10). The percentage of PNA‐positive seminiferous tubules at 21 days after injection was about 29.47 ± 3.2% (Figure 7d). Also, a comprehensive safety evaluation was conducted in *Maps* KO mice after mRNA delivery through histopathological analysis of multiple tissues throughout the body. Tissue specimens including heart, liver, spleen, lungs, kidney, and brain were systematically collected from both untreated *Maps KO* controls and rescued mice at 21 days after injection. Rigorous histological examination revealed no significant pathological alterations observed in the rescued tissues compared with *Maps* KO mice (Figure 7e). These findings demonstrated no obvious systemic toxicity associated with mRNA delivery in these mouse models. The successful rescue of meiotic arrest in these models validates the platform's potential for broad applicability in NOA‐affected patients with genetic disorders.

## 4 Discussion

**FIGURE 8:**
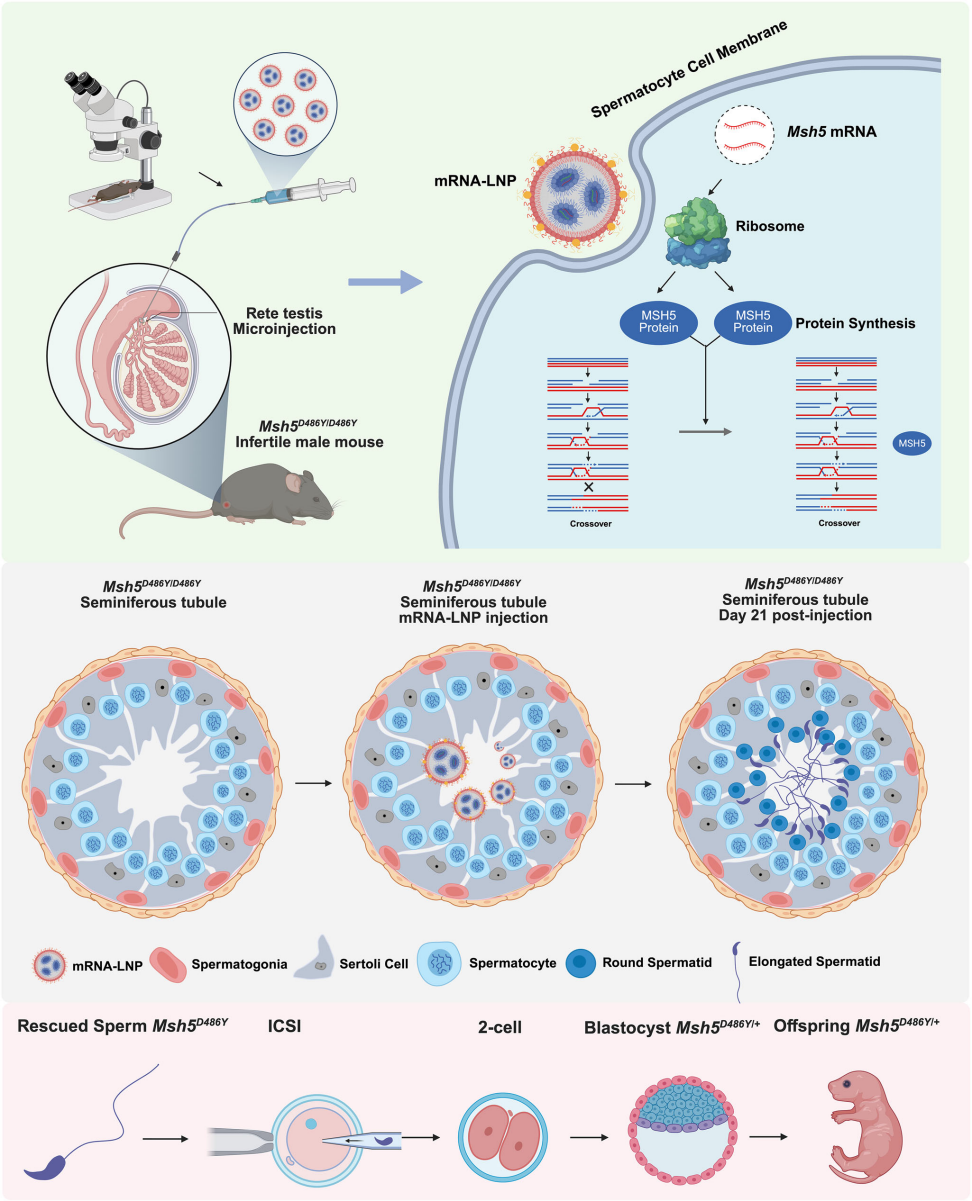
Schematic diagram of novel therapy for genetic spermatogenic disorder via testicular mRNA‐LNP delivery.

In the current study, we developed a novel therapy for genetic spermatogenic disorders. Herein, a spermatocyte‐tropic LNP (Pool1‐LNP3) was identified via a library of 30 ionizable lipids screening. And it was revealed that in vivo delivery of mRNA LNP3 could restore spermatogenesis in *Msh5^D486Y/D486Y^* and *Maps* KO mouse models with meiotic arrest. Notably, the offspring without genomic integration was born using ICSI derived from the rescue of *Msh5^D486Y/D486Y^* mouse and embryo transfer. Furthermore, no obvious inflammation and histologic damage in multiple tissues throughout the body were detected after in vivo delivery of LNP. Thus, we developed a novel testicular mRNA‐LNP delivery system as an effective solution for genetic spermatogenic disorders (Figure 8).

Prior attempts to address spermatogenic disorders with genetic defects have faced substantial limitations. Current therapeutic strategies for restoration of spermatogenesis in mice models with spermatogenic disorders derived from clinical NOA with genetic variants involved two CRISPR‐Cas9‐based approaches, including *ex vivo* editing of spermatogonial stem cells (SSCs) followed by testicular transplantation [41, 42, 43], and in vivo CRISPR/Cas9 delivery into seminiferous tubules for genetic correction [8]. Both strategies can restore spermatogenesis by correcting pathogenic variants, however, their clinical applications faced insurmountable barriers. Persistent concerns regarding CRISPR/Cas9 off‐target effects and incomplete validation of long‐term safety in vivo [44, 45, 46]. Notably, genome editing of human gametes and zygotes was strictly prohibited, rendering these approaches clinically inapplicable. Compounding these challenges, the absence of robust protocols for long‐term human SSCs culture in vitro further limits feasibility [47, 48]. In the current study, the novel mRNA‐LNP platform circumvents these limitations through a genomic non‐integrative mechanism. By delivering functional mRNA directly to spermatocytes, they transiently translated to wild‐type proteins in the cytoplasm, thus this approach achieves therapeutic protein expression without altering genomic integrity—a critical safety advantage over permanent genome editing.

RNA is a negatively charged molecule, and naked mRNA is unable to cross the cell membrane directly due to its charge properties, unless facilitated by techniques such as electroporation. However, while electroporation can enhance RNA delivery into cells, it induces obvious cellular damage and poses challenges for clinical applications. Additionally, RNA is highly susceptible to degradation by nucleases, which compromises its stability in vivo. Therefore, appropriate carriers should be used in the mRNA‐based therapeutic delivery due to their efficient delivery and biological stability. Previous studies have revealed that AAV vectors could be used to rescue spermatogenic disorders in mouse via delivery of therapeutic plasmids into seminiferous tubules [49, 50]. However, the clinical applications of AAV‐based therapies faced critical limitations in reproductive medicine. First, inherent risks of genomic integration, albeit at low frequencies, were identified in the AAV‐mediated plasmid delivery system, raising concerns about insertional mutagenesis in germ cells [51, 52]. Second, pre‐existing AAV DNA has been identified in human seminal plasma. It was detected in 35.9% (28/78) of men with abnormal semen parameters in a clinical cohort. This result indicated that AAV‐mediated plasmid delivery may compromise therapeutic efficacy through immune clearance, also suggesting potential associations between viral exposure and impaired spermatogenesis [53]. At last, AAV's constrained payload capacity (∼4.7 kb) limits its utility for the delivery of large genes [45]. In contrast, our LNP platform addresses these challenges comprehensively. As a non‐viral delivery system, LNPs eliminate risks of genomic integration while circumventing AAV‐related immunogenicity. Also, the safety of the mRNA delivery system was validated by the global administration of billions of LNP‐based COVID‐19 mRNA vaccines [54, 55]. The modular design of LNPs accommodates larger mRNA payloads, enabling delivery of full‐length therapeutic transcripts without size constraints. Furthermore, LNPs bypassed the complex manufacturing requirements of viral vectors, significantly enhancing scalability for clinical applications.

Current advancements in protein replacement therapies encompass diverse RNA modalities, including conventional mRNA, circular RNA (circRNA), and self‐amplifying RNA (saRNA). Notably, previous studies demonstrated that LNP with the cargo (saRNA encoding *DMC1*) could rescue spermatogenic arrest in *Dmc1* KO mice. The saRNA systems achieved prolonged intracellular protein expression through RNA replication (∼1 month). However, it was engineered with non‐structural protein elements derived from alphaviruses, raising concerns about the safety. Furthermore, endogenous *Dmc1* expression in wild‐type mice is tightly regulated, with mRNA and protein predominantly localized to spermatocytes during meiosis and absent in post‐meiotic spermatids. This pattern is conserved across key meiotic regulators in mammals, suggesting an advantage in safety of transient rather than sustained expression for rescue of spermatogenic disorders [56].

Additionally, rete testis microinjection was used to topical medication in our mRNA LNP delivery system, which facilitated the localization of mRNA‐LNPs in the lumen of the seminiferous tubule. Benefiting from the physiological characteristics of the blood‐testis barrier (BTB), the probability of mRNA‐LNPs spillover into systemic circulation is exceedingly low. This strategy is therefore designed to minimize ectopic expression in tissues beyond the testis. The rete testis microinjection method not only ensures the localized delivery of mRNA‐LNPs into the seminiferous tubules but also avoids the testicular tissue damage. Consequently, this technique holds greater potential for future clinical applications.

Although our study has demonstrated promising therapeutic efficacy in murine models, several limitations must be addressed to advance clinical application.

On the one hand, comprehensive validation of the platform's broad‐spectrum applicability requires testing in mouse models with different genetic defects. On the other hand, while our study identified the spermatocyte‐tropic LNP which could address meiotic arrest with genetic defects, spermatogenic arrest at spermatogonial or spermatid stages with genetic variants may necessitate distinct LNP delivery strategies. Further studies should be performed to develop spermatogonial stem cell/spermatid‐targeting platforms through either high‐throughput ionizable lipid screening (passive targeting) or antibody‐conjugated active targeting. In the current study, we demonstrated that spermatogenesis could be rescued using this delivery system in both *Msh5^D486Y/D486Y^* and *Maps* knockout mouse models. Notably, following embryo transfer, a live offspring was successfully obtained without genomic integration. However, a unique live birth was observed in the present study. In this study, the primary sequence of *Msh5* mRNA was chosen to be delivered into the seminiferous tubules. Nevertheless, there were lots of RNA modifications in *Msh5* endogenous mRNA (for instance, m6A, m1A, and m5C). Whether this RNA modification change could influence the expression of key regulatory factors in embryonic development warrants more in‐depth of investigation. Furthermore, the translation, protein folding, and the post translational modifications of exogenous mRNA may be not consistent to the endogenous mRNA. Thus, further studies should be carried out to evaluate the differences of the translation, protein folding and the post translational modification of exogenous and endogenous mRNAs and the effects of these distinct in the embryonic development. In addition, comprehensive investigations should be undertaken to systematically assess offspring size, weight, behavior, fertility, cognitive responses, and potential epigenetic alterations.

Collectively, our findings identified a novel testicular mRNA‐LNP delivery system as an effective solution for spermatogenic arrest with genetic defects. This evidence suggested broad clinical application potential of LNP‐based mRNA therapy as a safe and versatile solution for NOA‐affected patients harboring diverse genetic etiologies.

## Author Contributions

C.W.Z. carried out the experiments, data analysis, assisted with the experimental design, and wrote the manuscript. Nan Liang assisted with the design of the vector and the animal experiments. W.B.L., S.X., and P.L. carried out the experiments and data analysis. W.Z.N. and S.H. helped with immunofluorescence staining. Na Li provided technical help in flow cytometry analysis. N.J.O., E.L.Z., R.H.T., Y.H.H., and F.J.Z. provided technical help in rete testis microinjection. Y.F.S., H.W.B., J.P.Z., and X.J.B. helped with animal experiments. D.W.Q. helped with data analysis and statistics. F.R.B. assisted with ICSI and embryo transplantation. C.C.Y., Z.L., and H.C. conceived the project, supervised all experiments, wrote and revised the manuscript. All authors fulfill the criteria for authorship.

## Funding

This study was supported by the National Key R&D Program of China (Nos. 2022YFC2702700, 2022YFC2703004), National Natural Science Foundation of China (Nos. 82371607, 82371616, 82171590), Emerging advanced technology joint research project of SHDC (SHDC12023121), Shenzhen Science and Technology Program (20231120115406001 and 20240812083401001), and Pearl River Recruitment Program of Talents (2021QN02Y122).

## Conflicts of Interest

C.C.Y., C.W.Z., and Z.L. have applied for a patent based on this study. All other authors declare no potential conflicts of interest.

## Supporting information




**Supporting File 1**: advs74333‐sup‐0001‐SuppMat.docx.

**Supporting File 2**: advs74333‐sup‐0002‐SuppMat.docx.

**Supporting File 3**: advs74333‐sup‐0003‐MovieS2.mov.

## Data Availability

The data that support the findings of this study are available from the corresponding author upon reasonable request. All the relevant data supporting the key findings of this study are available within the article and its Supporting Information files or from the corresponding authors upon reasonable request. Also, the proprietary ionizable lipid (Pool1‐LNP3) can be obtained from the corresponding authors upon reasonable request.
