# Supplementary material for: Testicular mRNA‐LNP Delivery: A Novel Therapy for Genetic Spermatogenic Disorders
Source: Adv Sci (Weinh). 2026 Feb 11;13(22):e09855. doi: 10.1002/advs.202509855 (PMC13088321; doi:10.1002/advs.202509855)
Supplement: Supplementary file 1 — Supporting File 1: advs74333‐sup‐0001‐SuppMat.docx. [file ADVS-13-e09855-s003.docx]

**Fig S1. *In vitro* and *in vivo* validation of expression of *EGFP* mRNA-Pool 1, 2 and 3.** **(a, b, c)** Expression of EGFP after 2 μg transfection with *EGFP* mRNA- LNP Pool 1, 2 and 3 in 293T, TM3 and TM4 cells cultured in 6-well plates, scales bar = 20 μm. **(d, e, f)** Mean intensity of EGFP expression after 2 μg transfection with *EGFP* mRNA-LNP Pool 1, 2 and 3 in 293T, TM3 and TM4 cells cultured in 6-well plates, intergroup comparisons among the three groups were performed using the non-parametric Kruskal–Wallis test. Where the overall test indicated statistical significance, pairwise post hoc comparisons were conducted with Bonferroni-adjusted *P* values to control for type I error. Data were represented as median and interquartile range (IQR), n = 3 biologically independent mice per group. Significance thresholds: **P* < 0.05, ns: no significance.

**Fig S2. *In vivo* validation of expression of *EGFP* mRNA-LNP 1-10 in Pool1. (a)** *In vitro* validation of expression of EGFP after 2 μg transfection with *EGFP* mRNA-Pool1-LNP3 in 293T cells cultured in 6-well plates. **(b, c, d)** Immunofluorescence staining showed the expression of EGFP and the germ cell marker DDX4, the spermatogonia marker UCHL1 and the Sertoli cell marker Vimentin in testicular sections of Pool 1 LNP 1-10 (without LNP 3) after injection, NC denoted the negative control group administered with PBS, scale bar = 20 μm. **(e)** The percentage of EGFP-positive seminiferous tubules relative to the total seminiferous tubule population**,** intergroup comparisons among the three groups were performed using the non-parametric Kruskal–Wallis test. Where the overall test indicated statistical significance, pairwise post hoc comparisons were conducted with Bonferroni-adjusted *P* values to control for type I error. Data were represented as median and interquartile range (IQR), n = 3 biologically independent mice per group. Significance thresholds: ** *P* < 0.01, ns: no significance.

**Fig S3. Chemical structure of the ionizable lipid used in Pool1-LNP3.**

**Fig S4. Recovery of spermatogenesis of *Msh5^D486Y/D486Y^*male mice. (a)** Schematic diagram of *Msh5* mRNA + *EGFP* mRNA-LNP3 prepared by in vitro transcription and microfluidic systems. **(b)** Wholemount tissue fluorescence imaging of testis 7, 14 and 21 days after injection of *EGFP* mRNA-LNP3, white arrow head indicated the EGFP positive seminiferous tubules, scales bar = 5 mm. **(c)** Immunofluorescence staining showed PNA expression in *Msh5^D486Y/D486Y^* testis sections harvested in 7 days after injection of *Msh5* mRNA+*EGFP* mRNA-LNP3, scale bar = 20 μm. **(d)** Immunofluorescence staining showed the expression of PNA and TP1 in *Msh5^D486Y/D486Y^* testis sections harvested in 21 days after injection of *Msh5* mRNA+*EGFP* mRNA-LNP3, scale bar = 20 μm.

**Fig S5. The restoration of sperm chromatin and morphology in *Msh5^D486Y/D486Y^* mice caput. (a, b)** CMA3 staining and aniline blue staining of head condensation of spermatozoa from wildtype and *Msh5^D486Y/D486Y^* rescued mice testis and caput, scale bars = 20 µm. **(c)** Shorr's staining was used to assess the flagellar architecture of spermatozoa from wildtype and *Msh5^D486Y/D486Y^* mice, scale bars = 20 µm. **(d)** Immunofluorescence analysis of TOMM20 (green) and PNA (red) in spermatozoa from wildtype and rescued mice caput, scale bars = 10 µm.

**Fig S6.** **The restoration of spermatogenesis in *Msh5^D486Y/D486Y^* male mice**. Hematoxylin-eosin-saffron staining of *Msh5^D486Y/D486Y^* testis sections harvested in 14, 16, 18, 20, 22, 24, 26 and 28 days after injection of *Msh5* mRNA+*EGFP* mRNA-LNP3, yellow arrow indicated spermatocytes, blue arrow indicated round spermatids, green arrow indicated elongating, red arrow indicated elongated spermatids, scale bar = 50 μm.

**Fig S7. Timeline of rescued spermatids in *Msh5^D486Y/D486Y^* male mice after injection of *Msh5* mRNA+*EGFP* mRNA-LNP3.** Immunofluorescence staining showed the expression of PNA and TP1 in *Msh5^D486Y/D486Y^* testis sections harvested in 14, 16, 18, 20 and 22 days after injection of *Msh5* mRNA+*EGFP* mRNA-LNP3, scale bar = 20 μm.

**Fig S8.** **Chromosome spreading assay of *Msh5^D486Y/D486Y^* mice spermatocytes in day 3 post injection. (a)** Chromosome spreads analysis showed the expression of DMC1 foci and SYCP3 in spermatocytes with different stages in wildtype mice, *Msh5^D486Y/D486Y^* mice and *Msh5* mRNA-LNP3 treated *Msh5^D486Y/D486Y^* mice. **(b)** Chromosome spreads analysis showed the expression of SYCP1 (green) and SYCP3 (red) spermatocytes from wildtype mice, *Msh5^D486Y/D486Y^* mice and *Msh5* mRNA-LNP3 treated *Msh5^D486Y/D486Y^* mice, scale bar = 10 μm. **(c)** Chromosome spreads analysis showed the expression of MLH1 foci in spermatocyte at pachytene stage of wildtype mice, *Msh5^D486Y/D486Y^* mice and *Msh5* mRNA-LNP3 treated *Msh5^D486Y/D486Y^* mice, scale bar = 10 μm.

**Fig S9. Safety validation of *Msh5* mRNA in *Msh5^D486Y/D486Y^*.** Hematoxylin and eosin staining analysis showed revealed preserved tissue architecture without significantly pathological alterations in major organs (heart, liver, spleen, lungs, kidney, and brain) of *Msh5^D486Y/D486Y^* mice on day 21 after injection, scale bar = 50 μm.

**Fig S10. Recovery of spermatogenesis of *Maps* KO male mice.** Immunofluorescence staining showed the expression of TP1 and PNA in *Maps* KO testis sections harvested in 21 days after injection of *Maps* mRNA+*EGFP* mRNA-LNP3, scale bar = 20 μm.
