# Supplementary material for: Testicular mRNA‐LNP Delivery: A Novel Therapy for Genetic Spermatogenic Disorders
Source: Adv Sci (Weinh). 2026 Feb 11;13(22):e09855. doi: 10.1002/advs.202509855 (PMC13088321; doi:10.1002/advs.202509855)
Supplement: Supplementary file 2 — Supporting File 2: advs74333‐sup‐0002‐SuppMat.docx. [file ADVS-13-e09855-s002.docx]

Table S1. Primers for genotyping

| Primers | Sequence |
| --- | --- |
| *Msh5*-GT-F | 5’-CCCAAGGGATGAAAAGCCAC-3’ |
| *Msh5*-GT-R | 5’-GATACAGGGAGAGTAATGCGGTCTC-3’ |
| *Maps*-GT-F | 5’‐CCCGTTGCTCCGTGCATTTA‐3’ |
| *Maps*-GT-R | 5’‐TCTGCCTCCCGAGTGCTGTT‐3’ |

Table S2. Characterization of ionizable lipids and lipid nanoparticles

| LNP | Lipids (No.) | Mw | Size(nm) | PDI |
| --- | --- | --- | --- | --- |
| Pool1 |  |  |  |  |
| LNP-01 | 01 | 706.15 | 74 | 0.07 |
| LNP-02 | 02 | 720.18 | 77 | 0.06 |
| LNP-03 | 03 | 734.2 | 123 | 0.04 |
| LNP-04 | 04 | 804.34 | 77 | 0.13 |
| LNP-05 | 05 | 944.61 | 72 | 0.08 |
| LNP-06 | 06 | 818.32 | 76 | 0.09 |
| LNP-07 | 07 | 618.04 | 90 | 0.16 |
| LNP-08 | 08 | 702.21 | 79 | 0.18 |
| LNP-09 | 09 | 874.56 | 87 | 0.15 |
| LNP-10 | 10 | 688.14 | 85 | 0.15 |
| Pool 2 |  |  |  |  |
| LNP-11 | 11 | 720.18 | 96 | 0.08 |
| LNP-12 | 12 | 804.34 | 75 | 0.18 |
| LNP-13 | 13 | 860.45 | 92 | 0.13 |
| LNP-14 | 14 | 734.16 | 84 | 0.14 |
| LNP-15 | 15 | 702.21 | 91 | 0.15 |
| LNP-16 | 16 | 674.15 | 87 | 0.10 |
| LNP-17 | 17 | 702.21 | 90 | 0.19 |
| LNP-18 | 18 | 618.04 | 75 | 0.17 |
| LNP-19 | 19 | 589.99 | 88 | 0.13 |
| LNP-20 | 20 | 888.5 | 91 | 0.15 |
| Pool 3 |  |  |  |  |
| LNP-21 | 21 | 776.29 | 93 | 0.09 |
| LNP-22 | 22 | 706.11 | 76 | 0.09 |
| LNP-23 | 23 | 734.16 | 76 | 0.08 |
| LNP-24 | 24 | 660.13 | 83 | 0.17 |
| LNP-25 | 25 | 674.15 | 111 | 0.06 |
| LNP-26 | 26 | 604.02 | 94 | 0.18 |
| LNP-27 | 27 | 772.34 | 79 | 0.15 |
| LNP-28 | 28 | 604.02 | 90 | 0.17 |
| LNP-29 | 29 | 748.14 | 81 | 0.08 |
| LNP-30 | 30 | 748.23 | 91 | 0.18 |

Table S3. List of primary antibodies

| Antibody | Company | City, Country | Catalog Number | Host | Dilution | Clone Name | Reference |
| --- | --- | --- | --- | --- | --- | --- | --- |
| SYCP3 | Abcam^®^ | Cambridge, MA, USA | Ab97672 | Mouse | IF: 1: 200 | Cor 10G11/7 | ^1^ |
| SYCP3 | Abcam^®^ | Cambridge, MA, USA | Ab15093 | Rabbit | IF: 1: 200 | polyclonal | ^2^ |
| SYCP1 | Abcam^®^ | Cambridge, MA, USA | Ab15090 | Rabbit | IF: 1: 200 | polyclonal | ^3^ |
| DMC1 | homemade | - | - | Rabbit | IF: 1: 200 | - | ^4^ |
| MLH1 | BD Biosciences^TM^ | San Jose, CA, USA | 551092 | Mouse | IF: 1: 50 | G168-15 | ^3^ |
| DDX4 | Abcam^®^ | Cambridge, MA, USA | Ab13840 | Rabbit | IF: 1: 1000 | polyclonal | ^5^ |
| VIMENTIN | Servicebio^®^ | Wuhan, China | GB11192 | Rabbit | IF: 1: 200 | polyclonal | ^6^ |
| UCHL1 | Bio-Rad^®^ | Hercules, CA, USA | MCA4750 | Mouse | IF: 1: 500 | BH7 | ^7^ |
| EGFP | Abcam^®^ | Cambridge, MA, USA | Ab13970 | Chicken | IF: 1: 1000 | polyclonal | ^8^ |
| GFP | Invitrogen^TM^ | Waltham, MA, USA | A11122 | Rabbit | IF: 1: 1000 | polyclonal | ^9^ |
| AKAP3 | ProteinTech^®^ | Wuhan, China | 13907-1-AP | Rabbit | IF: 1: 200 | polyclonal | ^10^ |
| TP1 | ProteinTech^®^ | Wuhan, China | 17178-1-AP | Rabbit | IF: 1: 200 | polyclonal | ^11^ |
| PNA | Invitrogen^TM^ | Waltham, MA, USA | L32459 | - | IF 1:500 | - | ^12^ |
| TOMM20 | Abcam^®^ | Cambridge, MA, USA | Ab283317 | Mouse | IF 1:200 | EPR15581-39 | ^13^ |
| GFP | Youke^®^ | Shanghai, China | YKMP03120 | Mouse | WB: 1:1500 | - | ^14^ |
| ACTB-HRP | ProteinTech^®^ | Wuhan, China | HRP-66009 | Mouse | WB: 1:1000 | - | ^15^ |
| Hoechst33342 | Invitrogen^TM^ | Waltham, MA, USA | H21492 | - | IF 1:2000 | - | ^16^ |
| Alexa Fluor 488 goat anti-mouse IgG(H+L) | Invitrogen^TM^ | Waltham, MA, USA | A11001 | - | IF 1:1000 | - | ^17^ |
| Alexa Fluor 594 goat anti-mouse IgG(H+L) | Invitrogen^TM^ | Waltham, MA, USA | A11005 | - | IF 1:1000 | - | ^18^ |
| Alexa Fluor 488 goat anti-rabbit IgG(H+L) | Invitrogen^TM^ | Waltham, MA, USA | A11008 | - | IF 1:1000 | - | ^19^ |
|  |  |  |  |  |  |  |  |
| Alexa Fluor 594 goat anti-rabbit IgG(H+L) | Invitrogen^TM^ | Waltham, MA, USA | A11012 | - | IF 1:1000 | - | ^20^ |
| Alexa Fluor Plus 488 Goat anti-Chicken IgY (H+L) | InvitrogenTM | Waltham, MA, USA | A32931 | - | IF 1:1000 | - | ^21^ |

**Reference**

1 Jiang, X. *et al.* Maternal NAT10 orchestrates oocyte meiotic cell-cycle progression and maturation in mice. *Nat Commun* **14**, 3729 (2023). <https://doi.org/10.1038/s41467-023-39256-0>

2 Gainetdinov, I. *et al.* Relaxed targeting rules help PIWI proteins silence transposons. *Nature* **619**, 394-402 (2023). <https://doi.org/10.1038/s41586-023-06257-4>

3 Shimada, R. *et al.* STRA8-RB interaction is required for timely entry of meiosis in mouse female germ cells. *Nat Commun* **14**, 6443 (2023). <https://doi.org/10.1038/s41467-023-42259-6>

4 Hinch, A. G. *et al.* The Configuration of RPA, RAD51, and DMC1 Binding in Meiosis Reveals the Nature of Critical Recombination Intermediates. *Mol Cell* **79**, 689-701 e610 (2020). <https://doi.org/10.1016/j.molcel.2020.06.015>

5 Yang, W. *et al.* Zika virus disrupts steroidogenesis and impairs spermatogenesis by stalling the translation of CYP17A1 mRNA. *Nat Commun* **16**, 6756 (2025). <https://doi.org/10.1038/s41467-025-62044-x>

6 Yang, R. M. *et al.* Myeloid cells interact with a subset of thyrocytes to promote their migration and follicle formation through NF-kappaB. *Nat Commun* **14**, 8082 (2023). <https://doi.org/10.1038/s41467-023-43895-8>

7 Albariqi, M. M. *et al.* Human IAPP is a contributor to painful diabetic peripheral neuropathy. *J Clin Invest* **133** (2023). <https://doi.org/10.1172/JCI156993>

8 Feng, W. *et al.* ERG-driven prostate cancer initiation is cell-context dependent and requires KMT2A and DOT1L. *Nat Genet* **57**, 2177-2191 (2025). <https://doi.org/10.1038/s41588-025-02289-w>

9 Scholz-Carlson, E., Iyer, A. R., Nern, A., Ewer, J. & Fernandez, M. P. Synaptic targets of circadian clock neurons influence core clock parameters. *Sci Adv* **11**, eadw4666 (2025). <https://doi.org/10.1126/sciadv.adw4666>

10 Miyata, H. *et al.* SPATA33 localizes calcineurin to the mitochondria and regulates sperm motility in mice. *Proc Natl Acad Sci U S A* **118** (2021). <https://doi.org/10.1073/pnas.2106673118>

11 Pan, B. *et al.* Disrupted intercellular bridges and spermatogenesis in fatty acyl-CoA reductase 1 knockout mice: A new model of ether lipid deficiency. *FASEB J* **37**, e22908 (2023). <https://doi.org/10.1096/fj.202201848R>

12 Li, W. *et al.* Two-directional trafficking of the IFT25 protein in the developing mouse sperm flagella. *Biol Reprod* **112**, 309-318 (2025). <https://doi.org/10.1093/biolre/ioae171>

13 Liu, W. *et al.* VPS34 Governs Oocyte Developmental Competence by Regulating Mito/Autophagy: A Novel Insight into the Significance of RAB7 Activity and Its Subcellular Location. *Adv Sci (Weinh)* **11**, e2308823 (2024). <https://doi.org/10.1002/advs.202308823>

14 Luo, N. *et al.* Engineered ATG8-binding motif-based selective autophagy to degrade proteins and organelles in planta. *New Phytol* **237**, 684-697 (2023). <https://doi.org/10.1111/nph.18557>

15 Wrublewsky, S. *et al.* Heparan sulfate fine-tuned interleukin-1 (IL-1) signaling inhibits insulin secretion of grafted pancreatic islets. *Sci Adv* **11**, eady8566 (2025). <https://doi.org/10.1126/sciadv.ady8566>

16 Mittas, D. M. *et al.* Dual AAV vectors for efficient delivery of large transgenes. *Nat Protoc* (2025). <https://doi.org/10.1038/s41596-025-01243-8>

17 Gallardo, A. *et al.* BMAL1-TRIM28 represses transposable elements independently of CLOCK in pluripotent cells. *Nat Commun* **16**, 8250 (2025). <https://doi.org/10.1038/s41467-025-63778-4>

18 Hastar, N. *et al.* A pathogenic variant of AMOT leads to isolated X-linked congenital hydrocephalus due to N-terminal truncation. *J Clin Invest* **135** (2025). <https://doi.org/10.1172/JCI179438>

19 Walsh, C. M. *et al.* Localised delivery of interleukin-13 from a PLGA microparticle embedded GelMA hydrogel improves functional and histopathological recovery in a mouse contusion spinal cord injury model. *Bioact Mater* **53**, 855-874 (2025). <https://doi.org/10.1016/j.bioactmat.2025.07.018>

20 Xu, W. *et al.* Oxymatrine attenuates pulmonary fibrosis via APE1‑mediated regulation of the PINK1/Parkin pathway. *Mol Med Rep* **32** (2025). <https://doi.org/10.3892/mmr.2025.13627>

21 Rivera, J. F. *et al.* ATLAS: a rationally designed anterograde transsynaptic tracer. *Nat Methods* **22**, 1101-1111 (2025). <https://doi.org/10.1038/s41592-025-02670-x>
